# Self-assembled endogenous DNA nanoparticles for auto-release and expression of the *eGFP* gene in *Bacillus subtilis*

**DOI:** 10.1038/s42003-022-04233-8

**Published:** 2022-12-14

**Authors:** Linfeng Cao, Ziwen Meng, Junjie Tan, Ming Ying, Meiying Bi, Yanjun Liu, Xinrui Tong, Jiaxun Wei, Lei Huang

**Affiliations:** 1 Tianjin Key Laboratory of Organic Solar Cells and Photochemical Conversion, School of Chemistry and Chemical Engineering, Tianjin University of Technology, Tianjin, China; 2 Tianjin Key Laboratory of Drug Targeting and Bioimaging, School of Chemistry and Chemical Engineering, Tianjin University of Technology, Tianjin, China

**Keywords:** Nanostructures, Nanoparticles

## Abstract

The development of DNA delivery techniques is critical to promote the wider use of deoxyribonucleic acids as cellular transporters. The present study aimed to develop a type of DNA nanoparticle (*cit*Z-box) to automatically load and release cargo. The restriction enzyme can cleave *cit*Z-boxes at pro-designed sites, and the enhanced green fluorescent protein gene (*eGFP*) can be delivered into the *B. subtilis* protoplasts by them. The process of *eGFP* expression is recorded using a confocal microscope over 4 h. Here, multiscaffold and multimodular designs are used for *cit*Z-box assembly with a DAEDALUS module, DX_cage_design and rem (edge_length, 21), to ensure the structure was predicted as B-type DNA. Finally the *cit*Z-box is estimated to be a 50.7 nm cube. The 3D structure of the *cit*Z-box particle is detected to be approximately 50.3 ± 0.3 nm. DNA nanoparticles prepared as *cit*Z-boxes have great potential as drug carriers with automatic loading and releasing abilities.

## Abstract (web summary)

The DNA origami design algorithm DAEDALUS is modified to design a nanoscale cube with a two-step assembly protocol, assembling 9 modules from three DNA scaffolds and the complementary staples, combined to form the DNA origami cube.

## Introduction

Nucleic acids are suitable biomacromolecules to construct medicinal nanodevices^1–6^. However, nucleotide materials have similar problems to liposomal delivery systems, such as improving the uptake efficiency of cells and accurate auto-release^7^, among which the functions of auto-loading and release are critical to develop a DNA-based delivery platform. To achieve this goal, the present study aimed to design and assemble a type of DNA nanoparticle to deliver a gene, e.g., *eGFP*, to cells. To date, computer-aided DNA assembly approaches have focused on DNA tiles and origami. DNA tile design was the first method to use a set of short oligodeoxynucleotides to construct double crossover (DX) and triple crossover (TX) tiles, which must minimize the sequence symmetry to avoid possible undesired pairing and can self-assemble into 2D patterns^8–11^. DNA tile nanostructures, such as polypod-like structured and Y-shaped DNA, are generally produced by rolling circle amplification (RCA) using a single-stranded circular DNA as the template and enzymatic digestion^12–17^. However, organismal DNA is double-stranded and linear, and PCR technology is required for DNA nanotechnology to promote the wider use of this technology. By contrast, DNA origami, pioneered by Rothemund in 2006, can construct DNA nanostructures with almost any arbitrary shape and size using numerous short staple strands to fold into a long scaffold strand^18–21^. All the structures used one scaffold strand, for example, M13mp18, which required the synthesis of many different DNA strands, resulting in high synthesis costs^22–28^.

In this study, the modified DAEDALUS module, DX_cage_design, was used to design a 50.7 nm nanocube by reading in the sequence of *cit*Z gene, encoding citrate synthase of *B. subtilis* 168, and circulating it nine times with a remainder function, rem (edge_length, 21), ensuring to the predicted structure as B-type DNA^29^. The sequence of *cit*Z, used as scaffold strand, was folded nine times to form deoxynucleotide nanoparticles by multimodular self-assembly, named a *cit*Z-box. The scaffold strands were elongated to 9984 nt, which involved seven 1116 nt scaffolds and two short main scaffold strands of 812 nt and 1034 nt, respectively (Supplementary Fig. S1a). Atomic force microscopy (AFM), scanning electron microscopy (SEM), and transmission electron microscopy (TEM) revealed a nanocube with an average edge of 50.3 ± 0.3 nm, which was only 0.4 ± 0.3 nm different to the predicted size, which verified the feasibility of the modified software. To implement the auto-loading and release function, *BamH*I restriction enzyme sites were inserted into the two short scaffolds and used to construct a “can-lip” domain that is expected to load and encapsulate cargoes outside the cells, depending on sequence-specific cleavage. Sequence-specific bacterial endonucleases are suitable for developing DNA delivery platform, so long as the loaded DNA nanoparticles can be opened in cells, for example, by the restriction enzymes widely presenting in bacteria and archaea, or by non-specific mammalian endonucleases, such as DNase I and II. Fluorescence resonance energy transfer (FRET) demonstrated the automatic on-off performance of the “can-lip”. The fluorescence intensity of *Cy3* increased with increasing endonuclease concentration and disappeared when appropriate amounts of T_4_ DNA ligase were added. The delivery and auto-release functions of the *cit*Z-box were demonstrated using various plasmids with different copy numbers, resistances, and sizes, which were transformed into *B. subtilis* protoplasts with a more than tenfold increase in colonies. The whole process of “can-lip” opening was tracked using *Cy3*, recorded under a confocal microscope. Surprisingly, the expression of *eGFP* in *B. subtilis* protoplasts was observed within 4 h from delivery to release and expression. The results demonstrated that the DNA nanoparticles, designed and prepared as *cit*Z-boxes using endogenous DNA with restriction sites as a lip, have good potential as a drug-delivering platform.

## Results and discussion

### Construction of an endogenous DNA box

**Fig. 1:**
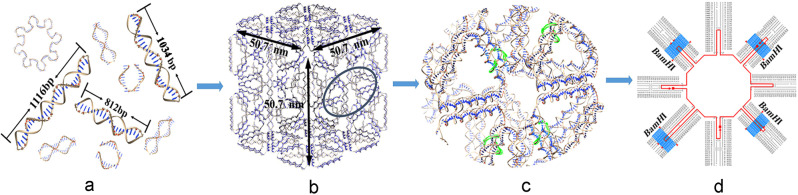
Diagram of the design framework for multiple scaffolds and restriction sites of *cit*Z-box assemblies. **a** Definition of the initial sequences of the endogenous gene (e.g., *cit*Z), its repeated use to specify the base sequences of multiscaffolds (for example: three lengths of main scaffold strands, 1116 bp, 812 bp, and 1034 bp) and staple strands (Scaf_*cit*Z_doubleXOVs.txt). **b** Prediction of *cit*Z-boxes with an edge of 50.7 nm by DAEDALUS, in which *BamH*I sites are circled with a black ellipse. **c** The amplification domain of restriction sites are marked with highlights. **d** The precise distribution of the region in which the*BamH*I sites (5′-GGATCC-3′) are inserted and highlighted.

To expand DNA nanotechnology to organismal DNA, we designed DNA nanocube (*cit*Z-box) using the sequences of *cit*Z (GeneID: 937381) from *B. subtilis* 168. We modified a Matlab open software package DEADALUS to predict a cube with three types of *cit*Z DNA strands: *Scaffold-M*, *Scaffold-2M*, and *Scaffold-9M* (Fig. 1a). According to the original algorithm of DAEDALUS, the stress of DNA folding is the smallest in B-type DNA molecules when each helix includes 10.5 base pairs; therefore, the length of each edge in the tetrahedral should round to the closest integer of N10.5 bp (N: positive integer more than 2). In this study, we added a remainder function, rem (edge_length, 21), to check whether the bases in each edge were a multiple of 21 bp, if not, they were extended by 5, 10, or 11 bases automatically. In addition, the output staple strands were as close as possible to [n10.5 bp], ensuring that the self-assembled nucleic acid does not bend excessively. The number of bases in a scaffold is unlimited in the DAEDALUS initial codes. To make the software suitable for ordinary DNA, the module of DX_cage_design was modified to read-in the sequence of *cit*Z, and the parameters were reset to circulate the *cit*Z gene nine times (9984 nt**)** (Supplementary Software). According to Eulerian circuit structure, the last cycle only requests 1056 nt to construct a closed symmetric cube, which required deletion 60 nt from the upstream and downstream of *cit*Z. To introduce the capability of automatic on-off, restriction sites of *BamH*I (5ʹ-GGATCC-3ʹ), which is not present in the original *cit*Z sequence, were inserted into the cube to form a lid (Fig. 1b). Therefore, the 1116 nt scaffold strand in the second cycle was split into a main scaffold, *Scaffold-2M*, with 812 nt, and some short strands with the alternative bases of 5ʹ-GGATCC-3ʹ, such as *Scaffold-2β*, *Scaffold-2γ*, and *Scaffold-2δ*. At the same time, the 1056 nt scaffold strand in the last cycle was also split into a short strand, *Scaffold-9α*, with the *BamH*I sites (Supplementary Table S1). There are four symmetrical *BamH*I restriction sites in same surface at the crossover of module II and IX of the *cit*Z-box (marked green in Fig. 1c). A single modular assembly method was used to synthesize nine modules separately, which were mixed to form the complete *cit*Z-box. The scaffold sequence of *cit*Z was stored in an array (scaf_seq), the bases of each edge of the cube were stored in a matrix (faces), and the bases of the vertices of the cube were stored in a sparse matrix (coordinates). The staple strands were designed according to complementary base pairing, and allocated to the corresponding edges according to the specified pattern.

To increase the stress of the *cit*Z-box, we designed the edges of the box containing two double-helixes. We rewrote the cube_fork. PLY file and designed the best location coordinates according to the spatial distribution of the *cit*Z-box structure.

**Fig. 2:**
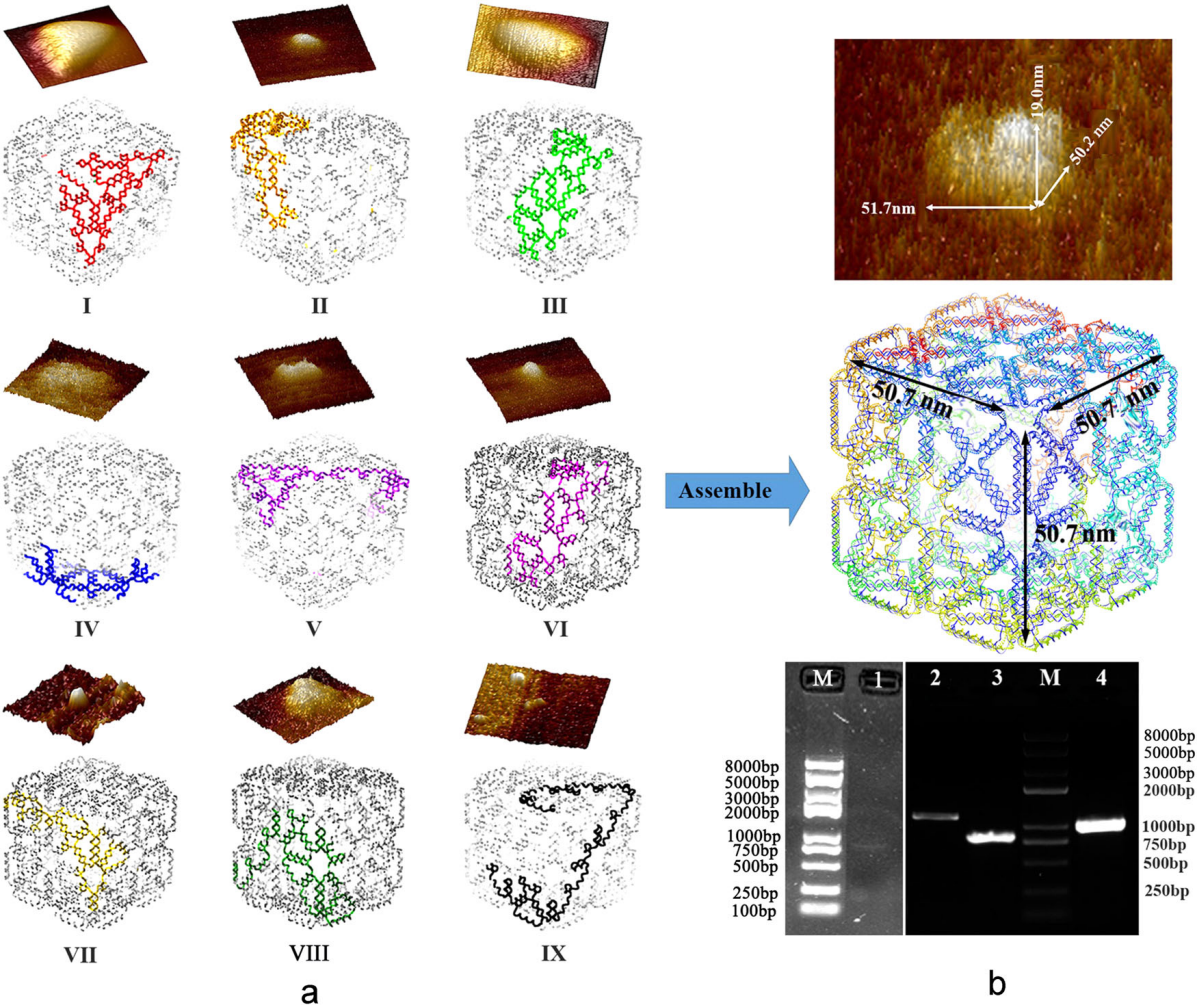
Diagram of the multimodular assembly. **a** The distributions of I–IX modules with *cit*Z-box structures and the corresponding AFM images. **b** The prediction and characterization of a complete*cit*Z-box analyzed using AFM (3D-size: 51.7 × 50.2 × 19.0 nm^3^) and agarose gel electrophoresis. 1: *cit*Z-boxes; 2: *Scaffold-M*; 3: *Scaffold-2M*; 4: *Scaffold-9M*; M: DNA marker.

Finally, the DEADALUS software output the corresponding staple strands file (Supplementary Tables S2–10), in which the four precise locations of *BamH*I sites can be found at G_1405_-C_1410_, G_1197_-C_1202_, G_1281_-C_1286_, G_8945_-C_8950_ (marked in blue in Fig. 1d). To obtain the atomic model of the designed DNA nanostructure, the software outputs the PDB file according to the convert_render_PDB.m program. According to the PDB file rendered by the Chimera software (https://www.cgl.ucsf.edu/chimera/), the different modules of the structure were rendered in different colors (Fig. 2a).

### Self-assembled multimodular *cit*Z-box

According to the software prediction, the *cit*Z-box nanoparticles should consist of nine modules, among them modules I and III–VIII have the same scaffold (1116 nt), module II has the multiscaffold in which *Scaffold-2M* is 812 nt, and the other short scaffolds,*Scaffold-2α*,*Scaffold-2β*,*Scaffold-2γ*, and *Scaffold-2δ* were designed to insert the *BamH*I site, and module IX has two scaffolds in which *Scaffold-9M* is 1034 nt, and *Scaffold-9α* is used to insert restriction sites (Supplementary Table S1). Thus, the modular assembly method was used to synthesize the *cit*Z-box. All scaffold amplifications used *B. subtilis* 168 genomic DNA as the templates for aPCR, except for the short strands, which were synthesized separately. The aPCR-amplified scaffolds were verified by agarose gel electrophoresis and stained by SybrSafe and were 1116 bp, 812 bp, and 1034 bp, in which the double-stranded DNA was the dominant product, probably caused by some undefined reasons, such as the used enzyme and primers, although aPCR is usually implemented to produce ssDNA (Fig. 2b and Supplementary Fig. S2). We think that aPCR is beneficial for the coexistence of ssDNA and dsDNA, which could be used as the scaffolds. In addition, a previous report used dsDNA as scaffolds to fold DNA origami successfully^30^. Therefore, we assembled the nine modules one by one using the double-stranded scaffolds initially. The bands at 750–1000 bp in the gel images were separated with the excess staples band ahead of them and purified for AFM observation (Supplementary Fig. S3a). AFM images of each module indicated the efficient formation of all the modules, because every block had a triangular structure similar to the expected size and shape (Fig. 2a). Second, the assembly solutions of each module were purified using a TIANgel Midi purification kit to remove the excess staple strands and some unused ssDNA. The purified products of each module, which were analyzed by Implen Microvolume Spectroscopy and agarose gel electrophoresis, were annealed directly in a thermocycler to assemble the cube structure. The purification yields of each module at the first step were 35–50% from module I to IX (Supplementary Table S11) and the gel images of the purified modules are shown in Supplementary Fig. S3b for comparison with the images before purification (Supplementary Fig. S3a). We speculated that the multimodules were successfully assembled preliminarily because the gel image of *cit*Z-boxes showed that the band migrated at 750–1000 bp different to the three scaffolds (Fig. 2b Lane 1, Supplementary Fig. S2 Lane 1 and Supplementary Fig. S3b Lane 10). We repeated the self-assembly experiments more than ten times. The efficiency of the self-assembly process was about 58.2 ± 0.2% and the *cit*Z-box concentration was 69.2 ± 0.2 ng/μL, as analyzed by Implen Microvolume Spectroscopy.

**Fig. 3:**
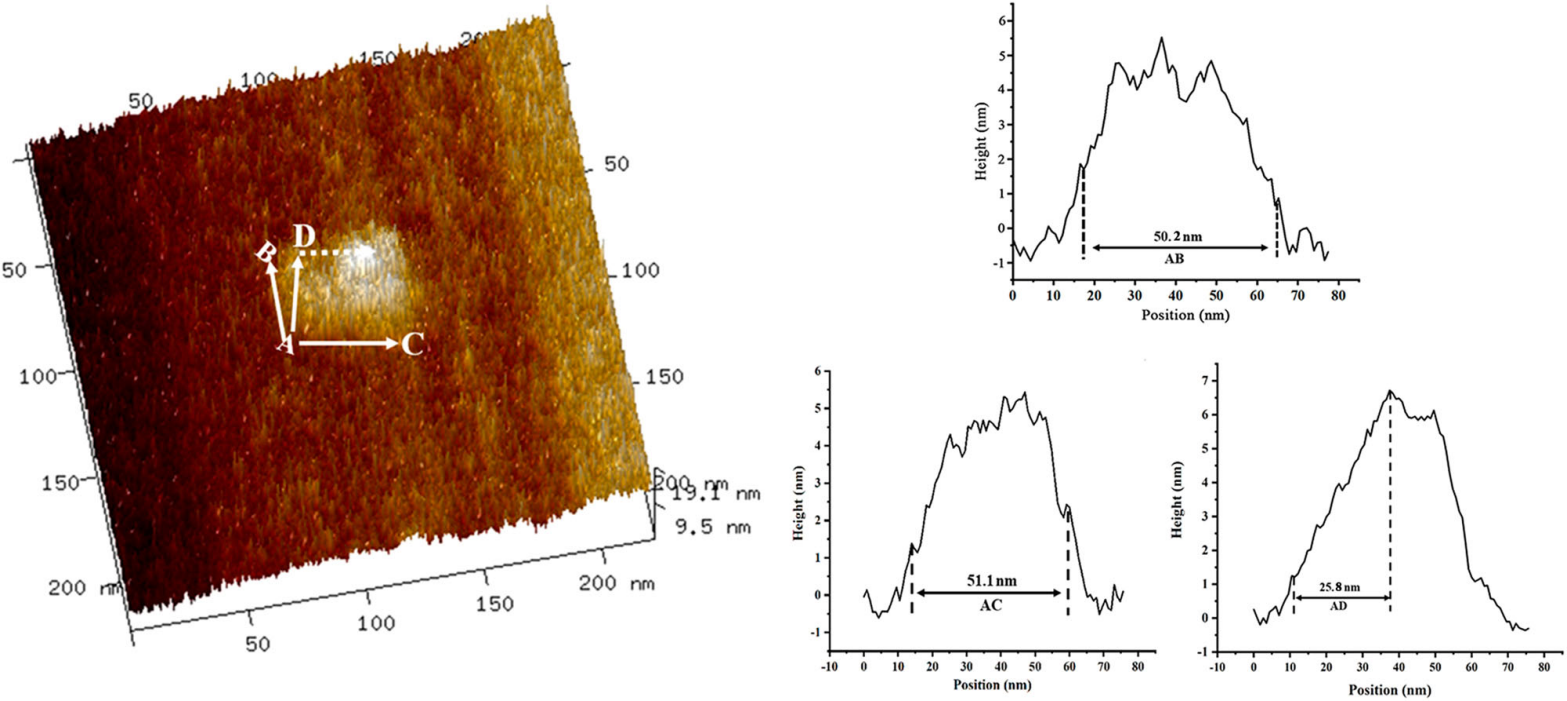
The 3D AFM image of a single *cit*Z-box particle with the corresponding height traces on the right. Lines A–B, A–C, and A–D show the height traces to the right. A–B: 50.2 nm, A–C: 51.1 nm, and A–D: 25.8 nm.

**Fig. 4:**
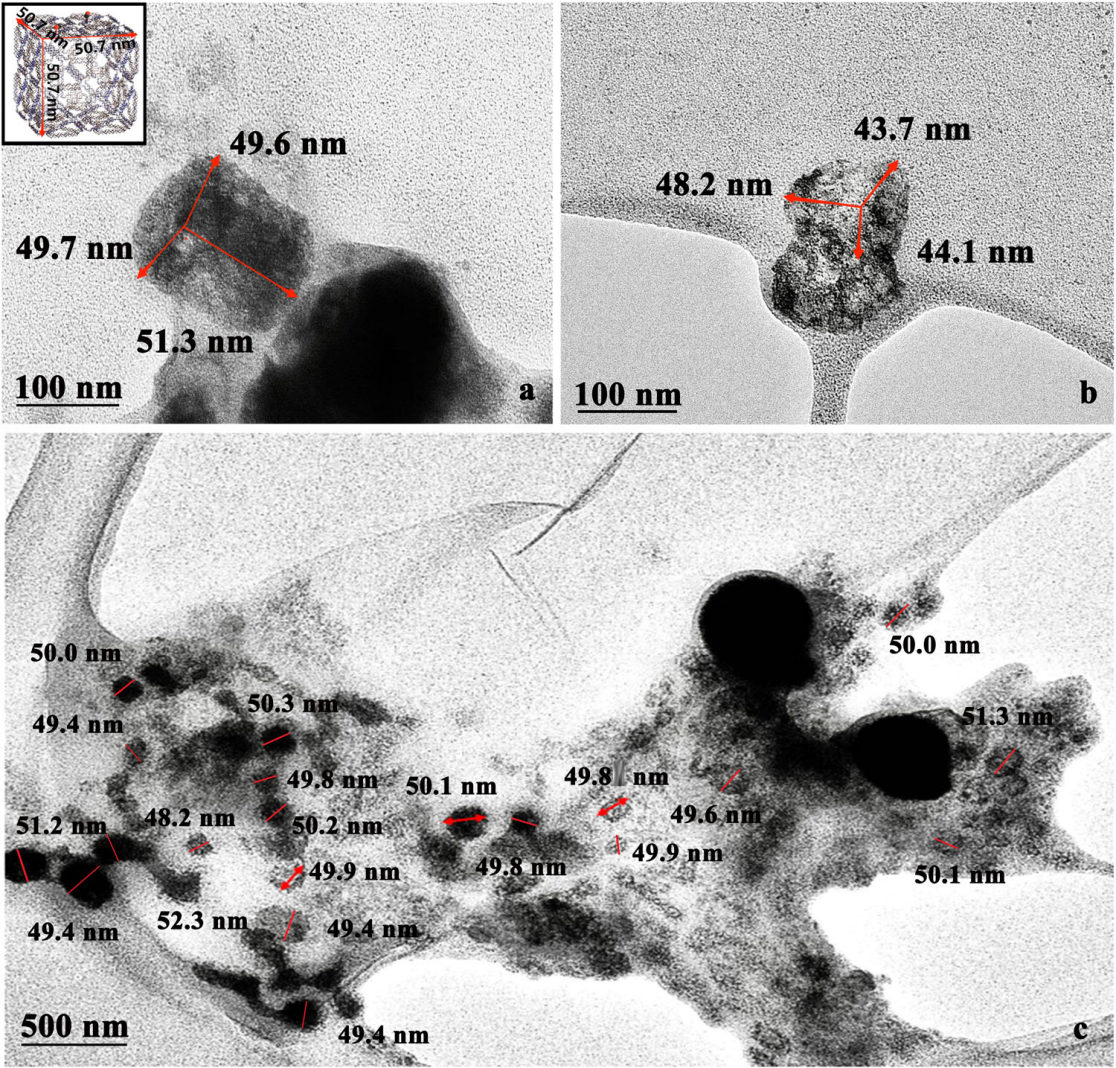
TEM images of a complete *cit*Z-box stained with the uranyl acetate. **a** An image of a single nanocube (3D-size: 49.7 × 49.6 × 51.3 nm^3^, Scale bar: 100 nm. **b** An image of two piled nanocubes (3D-size: 48.2 × 43.7 × 44.1 nm^3^), Scale bar: 100 nm. **c** Wide-field TEM (3 × 5 μm) image (average 3D-size: (50.5 ± 0.4) × (50.0 ± 0.4) × (50.3 ± 0.2) nm^3^). Data are expressed as mean ± s.d. *n* = 20 independent samples. Scale bar: 500 nm.

Multimodular self-assembly was also verified using AFM. The image of the surface morphology in Fig. 2 showed a distinctive cube structure although the samples have a certain degree of deformation because of the probe pressure caused by the automatic tap scanning mode, which resulted in the height of the sample being only 19.0 nm, whereas the length and width were 51.7 nm and 50.2 nm, respectively, which were consistent with the pro-design (50.7 nm^3^) (Fig. 2b). For detailed analysis, we select a different AFM image to define size of the *cit*Z-box with the height traces (Fig. 3). However, again the needle insertion force deformed the height of the particles from A to D to about 25.8 nm, while only a small deformation occurred in length from A to C and width from A to B, of about 0.5 nm, in accordance with other measured results (Supplementary Data 1). The morphology of the *cit*Z-boxes was also characterized by TEM after staining with uranyl acetate solution. Some distinctive nanocubes are shown in Fig. 4a, b with ~50 nm^3^ three-dimensional size without deformation and are similar to the pro-design.

**Fig. 5:**
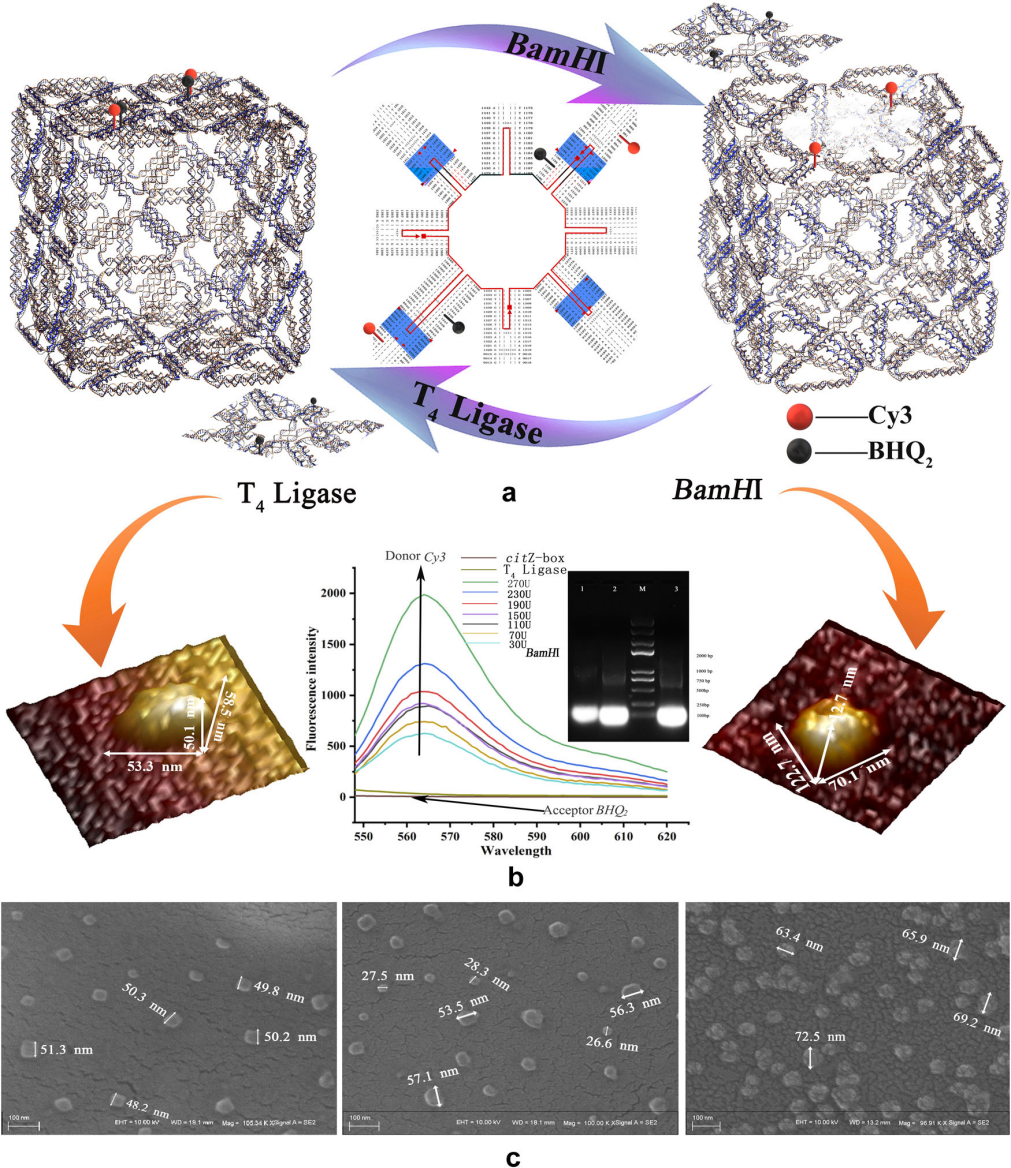
The “can-lid” design and characterization of *cit*Z-box treated with *BamH*I/T_4_ ligase. **a** Diagram of the *cit*Z-box treated with *BamH*I/T_4_ ligase that was labeled by *Cy3* and *BHQ*_*2*_. **b** The AFM images of *cit*Z-boxes treated with T_4_ ligase and *BamH*I. FRET with different concentrations of *BamH*I and T_4_ ligase and gel electrophoresis diagrams.1: normal *cit*Z-box; 2: a *cit*Z-box opened by *BamH*I; M: DNA Marker, 3: a *cit*Z-box closed with T_4_ligase. **c** SEM images of the complete *cit*Z-box (average size: 50.0 nm), the particles digested by *BamH*I (average size: 55.7 nm) and then treated with T_4_ ligase (average size: 67.8 nm). Scale bar: 100 nm.

Moreover, SEM was used to further determine the spatial conformation of a large number of *cit*Z-boxes (~300). The results showed many uniformly dispersed and regular nanocube structures (Fig. 5c). The average size of the samples calculated from dozens of wide-field SEM images (0.8 × 1μm), selecting twenty particles per image, was about 50.3 ± 0.2 nm, which was only 0.4 ± 0.2 nm different to the software-predicted dimension. The three-dimensional sizes of the *cit*Z-boxes were also analyzed using dozens of the wide-field AFM (2 × 2 μm) and TEM (3 × 5 μm) images, selecting twenty particles per image, which were (50.5 ± 0.4) × (50.0 ± 0.4) × (50.3 ± 0.2) nm^3^ (Fig. 4c).

Taken together, the results showed the *cit*Z-box nanoparticles were assembled successfully using dsDNA as scaffolds. We speculated that the complementary strands of the dsDNA scaffolds would probably be substituted by the corresponding staples and separated away during individual module purification, such that reverse complementation did not happen when assembling the entire cube. However, we think the approach used for the successful assembly *cit*Z-box, i.e., the multiscaffold and multi-module protocol, would be suitable for using dsDNA as materials.

### Restriction endonucleases open the *cit*Z-box

Restriction endonucleases are endogenous enzyme distributed in bacteria; therefore, restriction sites were inserted into the second and the ninth modules of the *cit*Z-box, which could be cleaved by endonucleases to automatically open the box. This would enable *cit*Z-boxes to load drugs in vitro and auto-release them by sequence-specific or non-specific endonucleases, such as DNase I and II, in mammalian cells. In the following experiment, the modified scaffold strands *Scaffold-2β*, *Scaffold-2γ*, *Scaffold-2δ*, and *Scaffold-9α* with *BamH*I sites in modules II and IX replaced the partial sequences of the original *cit*Z gene. A “can-lid” sheet will be cut off by *BamH*I from the surface composed of modules II and IX, leaving a hole in the same position, similar to a screwdriver opening a can-lid. The “can-lid” can also be sealed again with DNA T_4_ ligase after loading (Fig. 5a). Agarose gel electrophoresis was used to verify this process, the results in Fig. 5b showed the difference in electrophoretic mobility before and after *BamH*I digestion. The samples digested by *BamH*I migrated faster than the normal box because its structure was no longer tightly packed together resulting in less electrophoretic resistance; however, the samples treated by T_4_ ligase migrated more quickly because the structure could not return to its original conformation, becoming larger and looser (Fig. 5b and Supplementary Fig. S4). This speculation was confirmed by AFM and SEM observations. The particles in *cit*Z-box samples digested by *BamH*I were slightly bigger than the original at 55.7 nm, but were uniformly distributed in the second SEM image, possibly because the topology of the DNA double helix had changed. We observed some smaller square particles with an average size of 27.5 nm, which represented the “can-lid” domains separated from the *cit*Z-boxes (Fig. 5c). The third SEM image shows the T_4_ ligase-treated samples, which were larger than those after the digestion reaction (69.8 nm). Moreover, the square particles of 20–30 nm disappeared, illustrated the *cit*Z-boxes were sealed by T_4_ ligase. The corresponding AFM images showed, in Fig. 5, the size of particle was changed to 122.7 × 70.1 × 12.7 nm^3^ and there was an obvious dimple on the surface. After sealing with T_4_ ligase, the particle size was restored to 58.5 × 53.3 × 50.1 nm^3^, which was also slightly larger and consistent with the results of SEM (Fig. 5b).

Next, we used *Cy3* fluorophores to label the bases C_8933_ and A_1001_, and correspondingly inserted *BHQ*_*2*_ quenching group at the positions A_1346_ and T_1194_ (Fig. 5a). The absorption spectrum of *BHQ*_*2*_ is 560–570 nm, allowing it to absorb the emissions of *Cy3* (em: ~570 nm). If the two groups are close enough, the distance of *Cy3* between *BHQ*_2_ was only 13–15 bp (less than 10 nm) in *cit*Z-box$^{Cy3{\mbox{-}}BHQ2}$, which cannot be detected by the Hitachi F-7000 fluorescence spectrophotometer because the emissions of *Cy3* are absorbed by *BHQ*_*2*_ perfectly. After *BamH*I digestion, the *Cy3* fluorophore remained on the *cit*Z-box^*Cy3-BHQ2*^, and the quencher group *BHQ*_*2*_ was separated, increasing the distance between *Cy3* and *BHQ*_*2*_ and the fluorophore *Cy3* would emit red fluorescent ~570 nm when excited using the 532 nm laser. This phenomenon is displayed by the fluorescence (FL) spectra in Fig. 5b, demonstrating that *cit*Z-box^*Cy3-BHQ2*^ can be cleaved gradually after the addition of *BamH*I: The samples emitted ~570 nm fluorescence and the intensity increased from 500 to 2000 when *BamH*I was increased from 30 U to 270 U, i.e., the more *Cy3* fluorescent groups separated, the stronger of the fluorescence. After the addition of T_4_ ligase, the fluorescence disappeared (Fig. 5b and Supplementary Data 2). This experiment proved that *cit*Z-box can be automatically opened by *BamH*I and closed by T_4_ ligase in vitro.

### Auto-release and expression of *eGFP* in *B. subtilis* protoplasts

**Fig. 6:**
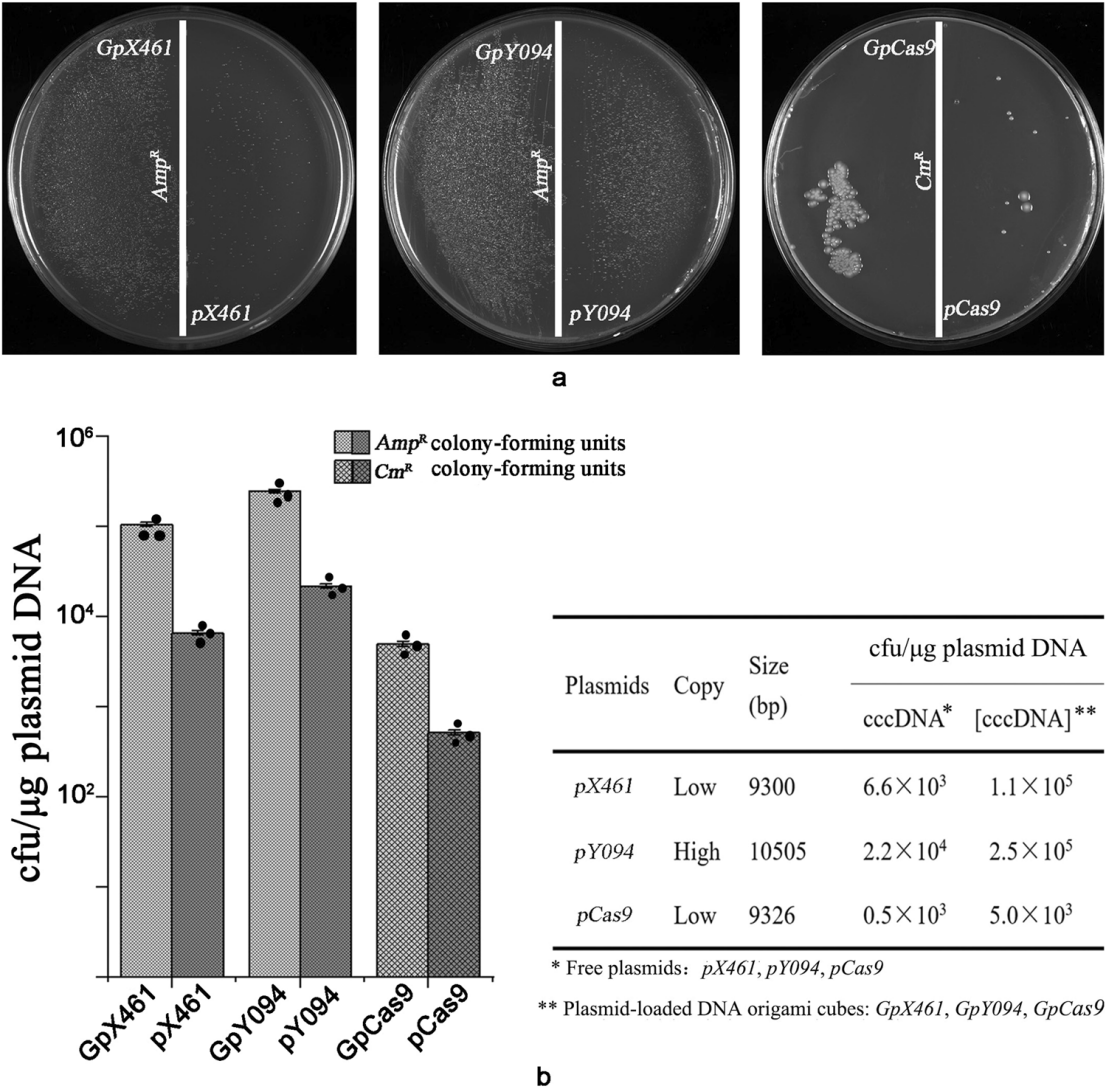
Characterization of *cit*Z-boxes delivering plasmids into *B. subtilis* 168 protoplasts. **a** Comparisons of transformation experiments for *GpX461*, *GpY461*, *GpCas9* and *pX461*, *pY094*, and *pCas9*. **b** Histogram and statistics of the colony formation of each comparison. Data are expressed as mean ± s.d. *n* = 3 independent experiments.

We hoped that the *cit*Z-boxes could be used as cellular shuttles to load and release inclusions freely. To confirm this, *B. subtilis* 168 were used as the target cells and prepared as protoplasts, which can easily engulf external substances. Three types of plasmids, *pCas9* (9326 bp, *Cm*^*R*^) *pX46*1 (9288 bp, *Amp*^*R*^, *eGFP*), and *pY094* (10463 bp, *Amp*^*R*^, *eGFP*) were selected to test the performance of the *cit*Z-boxes (Supplementary Fig. S1b). The three plasmids were loaded into *cit*Z-boxes through the reactions of *BamH*I and T_4_ ligase, and constructed as *GpCas9*, *GpX461*, and *GpY094*, respectively, which were transformed into *B. subtilis* 168 protoplasts. The free plasmids, *pCas9*, *pX461*, and *pY094*, were also transformed simultaneously. It is difficult to characterize the plasmid loading efficiency for *cit*Z-boxes, which might be further explored and reported in the future. We designed the protoplasts transfection experiments with the modified method of Chang and Cohen (1979) according to Addgene’s corresponding plasmid protocol. Luria–Bertani agar with 25 μg/mL chloramphenicol was used to select *GpCas9*/*pCas9* transformants, while ampicillin at 100 μg/mL was used for *GpX461*/*pX461*and *GpY094*/*pY094*. The number of colonies on Luria–Bertani agar were obviously different between the free plasmids and plasmid-loaded *cit*Z-box groups. There were markedly more colonies formed by the plasmid-loaded *cit*Z-boxes than those formed by the free plasmids, regardless of their size, copy number, and resistance gene. Although chloramphenicol represses protein synthesis, thus forming fewer colonies when transformed with *GpCas9*, more colonies were formed compared with using free *pCas9* (Fig. 6a). In addition, the experiments also showed that the resistance genes were expressed normally from the plasmids, indicating the *cit*Z-boxes were opened autonomously by the host cells. The results in the column diagram and data sheet indicated that the number of colonies induced by free plasmid *pX461* carried in *cit*Z-boxes increased from 6.6 × 10^3^ to 1.1 × 10^5^ cfu/μg plasmid DNA (~17-fold), and that of *pY094* vs. *GpY094* and *pCas9* vs. *GpCas9* increased from 2.2 × 10^4^ to 2.5 × 10^5^ cfu/μg plasmid DNA, and 0.5 × 10^3^ to 5.0 × 10^3^ cfu/μg DNA(~tenfold), respectively (Fig. 6b and Supplementary Data 3). The free plasmids *pCas9*, *pX461*, and *pY094* were used in the transformation experiments at the same concentration as that added into the loading system. Thus, we speculated that the increase in the number of colonies was caused by the improvement of transformation efficiency by loading with *cit*Z-box. However, it is currently very difficult to characterize the plasmid number loaded per *cit*Z-box, thus the improved transformation efficiency induced by the *cit*Z-boxes still needs to be verified in further research.

**Fig. 7:**
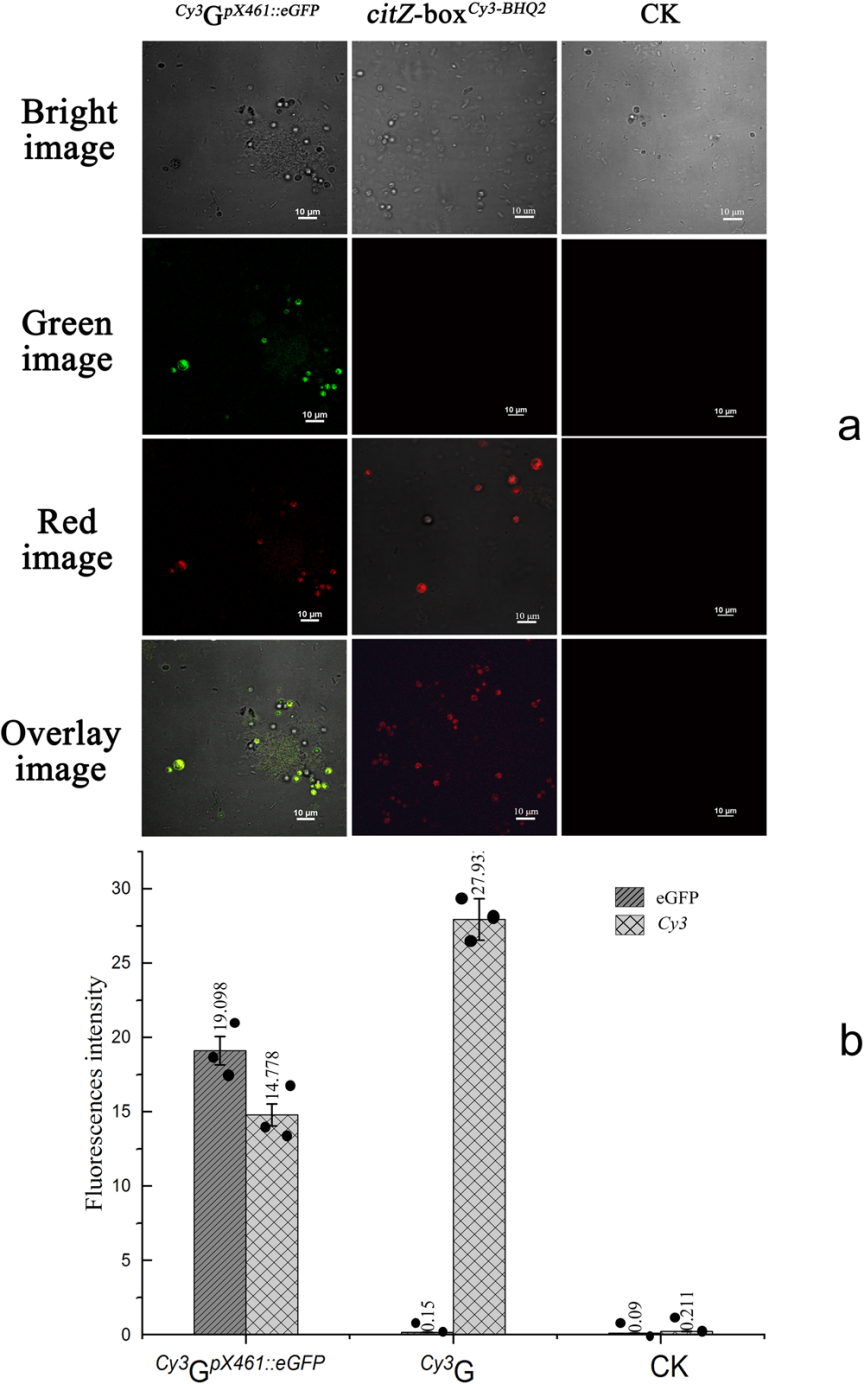
CLSM analysis of protoplasts of *B. subtilis* 168 transformed with ^*Cy3*^G^*pX461::eGFP*^ (*cit*Z-box^*Cy3-BHQ2*^ loaded with *pX461*, *Amp*^*R*^, and *eGFP*), *cit*Z-box^*Cy3-BHQ2*^ (*cit*Z-box labeled by *Cy3* and *BHQ*_*2*_ without *eGFP*) and untransformed cells under different excitation conditions. **a** CLSM images of protoplasts excited by 543.5 nm and 488.0 nm lasers to detect *Cy3* and eGFP, respectively. Scale bar: 10 μm. **b** A graph of real-time fluorescent intensity. Data are expressed as mean ± s.d. *n* = 3 independent experiments.

To explore the activities of *cit*Z-boxes in cells, we transformed *B. subtilis* 168 protoplasts with ^*Cy3*^G^*pX461*::*eGFP*^, comprising *citZ-box*^*Cy3-BHQ2*^ carrying plasmid *pX461* with *eGFP*. If the *cit*Z-box enters the host cell and is automatically opened, red *Cy3* fluorescence would be emitted at an excitation wavelength of 543.5 nm. The successful release of the plasmid and *eGFP* expression would be indicated by green fluorescence at an excitation wavelength of 488.0 nm. This experimental route is described in detail in an animation file (Supplementary Movie 1). A Nikon A1 confocal laser-scanning microscope was used to track ^*Cy3*^G^*pX461*::*eGFP*^ in cells. After ^*Cy3*^G^*pX461*::*eGFP*^ was transformed into protoplasts and incubated for 60 min, the cells emitted red fluorescence at an excitation wavelength of 543.5 nm and green fluorescent at 488 nm (Fig. 7a). The real-time fluorescent intensity could be detected at the same time (Fig. 7b), while the control of untransformed cell (CK) did not show any green/red fluorescence when excited by 543.5 nm or 488 nm, and the transformed cells with *cit*Z-box^*Cy3-BHQ2*^ without eGFP emitted red fluorescent but no green fluorescent (Supplementary Data 4). This phenomenon indicated that ^*Cy3*^G^*pX461::eGFP*^ entered the host cell and was opened automatically resulting in successful *eGFP* expression. We believe that *B. subtilis* 168 easily distinguished the *cit*Z gene sequence and absorbed it actively. Thus, endogenous deoxyribonucleotide nanomaterials would be biocompatible.

**Fig. 8:**
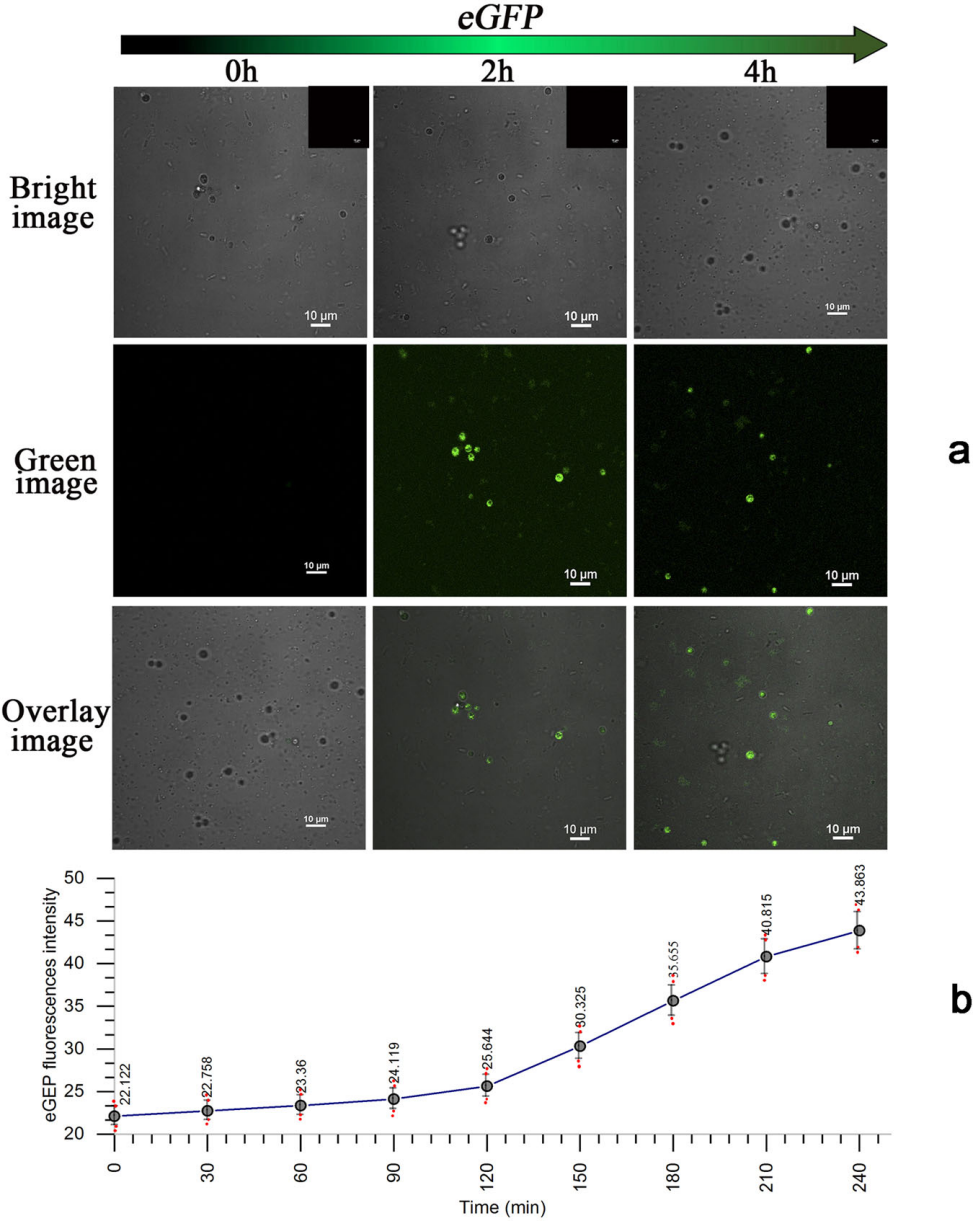
CLSM analysis of different protoplasts of *B. subtilis* 168 at 2-h intervals, which were transformed with *GpY094* (*cit*Z-boxes loaded with *pY094*, *Amp*^*R*^, and *eGFP*) and incubated in CMR for more than 4 h. **a** CLSM images of protoplasts excited using *λ*_ex_/*L*_in_of 488.0/500–530 nm to detect eGFP. Scale bar: 10 μm. **b** A graph of real-time fluorescent intensity over 4 h. Data are expressed as mean ± s.d. *n* = 3 independent experiments.

During *cit*Z-box protoplast transformation, we observed the whole process of *eGFP* expression using CLSM. To verify that *cit*Z-boxes can automatically release the inclusions in cells, *GpY094*-loaded *cit*Z-boxes were transferred into protoplasts and tracked in living cell culture device using CLSM for more than 4 h. The 4 h fast-forwarded video recorded the green fluorescent protein encoded by *eGFP* carried by the *pY094* plasmid flowing out of the *cit*Z-boxes (Supplementary Movie 2), in which we observed the globular cells emitting green fluorescence, which increased gradually with time. Plasmid *pX461* does not have an independent replicator and homology gene of *B. subtilis* 168, and thus is not inherited and is only transcribed at the first generation. However, we could observe the entire process of *eGFP* expression in the first generation of *B. subtilis* 168. Moreover, with increasing time, the fluorescence of the sample became stronger. When *GpY094* transformation was terminated using SMMP, only a few cells with green fluorescence were detected, suggesting that the *eGFP* gene might be expressed only initially. However, 2 h later, the numbers of green fluorescent cells and the fluorescence intensity increased gradually; and the non-fluorescent rod-shaped cells appeared at the same time. At 4 h of incubation, the fluorescent intensity of the cells was basically the same as that at 2 h, and the number of green cells had not increased; however, the number of non-fluorescent rod-shaped had increased, suggesting that the cells had restored their nutritional status and started reproduction (Fig. 8a). The changes of fluorescent intensity with time were recorded throughout the process (Fig. 8b and Supplementary Data 5). The fluorescent signals became stronger after 2 h of transformation. After 4 h, the green fluorescence gradually disappeared, possibly because of the dilution of *eGFP* caused by cell division. Thus, the gene of *eGFP* was delivered and auto-released for successful expression in *B. subtilis* by the *cit*Z-boxes.

### Conclusion

In this study, the *B. subtilis* 168 *cit*Z gene was used to construct a cube with an edge of 50.7 nm and the nanocube particles, *cit*Z-boxes, were assembled using multiscaffolds and modules. We demonstrated the feasibility of using endogenous double-stranded DNA as materials for DNA nanotechnology, which formed a facile and reliable carrier for the host cells. The endogenous DNA sequences were more easily recognized and accepted by the organism, resulting in 10–20-fold increases in the cfu of plasmids carried by *cit*Z-boxes. We further speculated that the transformation efficiency was probably improved. However, due to the limited characterization of time and plasmid loading efficiency of the DNA origami cube, it remains a difficult problem in the industry at present, which will requires us to design experiments, the results of which will be published in a future study, although this might take a long time. The *cit*Z-box particles, containing inserted *BamH*I restriction sites, were opened by endonucleases, as confirmed by FRET of *Cy3-BHQ*_*2*_ in vitro and the auto-release of *eGFP* in *B. subtilis* protoplasts. We observed the expression process of *eGFP* transport by *cit*Z-boxes using CLSM during the first 4 h, which indicated that *cit*Z-boxes can automatically release their inclusions into cells, where they might be cleaved by the corresponding endonuclease or other enzymes, such as DNase I and II, whether in bacterial or mammalian cells. We believe that this assembly idea will have wide applications in targeted delivery to bacteria for combating infectious diseases and will lead to the development of programmable DNA nanostructures.

## Methods

### Software improvement

The software package used for *cit*Z-box design was DAEDALUS, developed by the Laboratory of Computational Biology and Biophysics, MIT (MA, USA), which is open-source software under the GNU General Public License, version 2 (GPL-2.0) and is available at http://daedalus-dna-origami.org. DAEDALUS runs on MATLAB and can render almost any three-dimensional DNA structure with single-stranded DNA (M13mp18, 7249 bp). According to the software instructions, (1) the module DX_cage_design was rewritten to read in the sequence of *cit*Z; (2) the sequence of *cit*Z was used repetitively as a multiscaffold nine times, involving eight 1116 nt and one 1056 nt sequence; and (3) the coordinates of the PLY file were revised to generate a cube_fork model. Then, the PDB file of *cit*Z-box was automatically outputted to provide the coordinates of each atom. The sequences of the scaffold and staple strands were outputted and are listed in the Scaf_*cit*Z_doubleXOVs.txt file (Supplementary Software).

### Preparation of multiscaffolds

The endogenous DNA, the *cit*Z gene, used as multiscaffold, was prepared using a modified asymmetric PCR (aPCR) reaction^31^, with the genome of *B. subtilis* 168 (NCBI accession no: NC_000964.3) as the template (Table 1), which was extracted and purified using a TIANamp bacteria DNA kit purchased from TIANGEN Biotech (Beijing, China). The primers, *cit*Z-M-F and *cit*Z-M-R, *cit*Z-II-F and *cit*Z-II-R, and *cit*Z-IX-F and *cit*Z-IX-R (Table 1), were used to amplify the *cit*Z-box scaffolds, *Scaffold-M* (1116 bp), *Scaffold-2M* (812 bp), and *Scaffold-9M* (1034 bp), respectively (Supplementary Table S1). The aPCR reaction system included 1.0 nM sense primer, 1.5 nM antisense primer, 20 ng of *B. subtilis* 168 genomic DNA, 10 μM dNTP, 0.25 μL (5 U/μL) of Taq DNA polymerase and ddH_2_O to a final volume of 50 μL. A Biometra Tgradient thermocycler (Jena, Germany) was used for scaffold amplification with the procedures of template denaturation at 94 °C for 4 min; followed by 30 cycles of 94 °C for 1 min, 60 °C for 50 s at 72 °C 1.5 min for elongation; and a final incubation at 72 °C for 5 min. The amplicons were stored at 4 °C. Taq DNA polymerase, oligonucleotides, and other agents used for PCR were purchased from Takara Biotech (Dalian, China) and Ruibio Biotech (Beijing, China).

**Table 1:** Oligo sequences, strains, and plasmids used in this study.

Strain			Characteristics	Source (usage)
*Escherichia coli* DH5α			Host for *pCas9*, *pX461*, and *pY094*	Laboratory stock
*Bacillus subtilis* 168			Gram-positive bacteria, engineering bacteria	Laboratory stock
**Free plasmids and plasmids-loaded** ***cit*Z-box**				
*pCas9*	Bacterial expression of Cas9 nuclease, tracrRNA and crRNA guide, Chloramphenicol, 25 μg/mL.			Addgene http://www.addgene.org/42876/
*pX461*	Cas9n (D10A nickase mutant) from *S. pyogene*s with 2A-*eGFP*, and cloning backbone for sgRNA Ampicillin, 100 μg/mL			Addgene http://www.addgene.org/48140/
*pY094*	Expresses huAsCpf1-T2A-*GFP* and crRNA guide Ampicillin, 100 μg/mL			Addgene http://www.addgene.org/84743/
*GpCas9*	*cit* Z-box loaded with*pCas9*,*Cm*^*R*^			This work Addgene ID 194080
*GpX461*	*cit* Z-box loaded with*pX461*,*Amp*^*R*^ *eGFP*			This work Addgene ID 194081
*GpY094*	*cit* Z-box loaded with*pY094*,*Amp*^*R*^*, eGFP*			This work Addgene ID 194082
^*Cy3*^*G*^*pX461*::*eGFP*^	*citZ-box*^*Cy3-BHQ2*^ loaded with *pX461,Amp*^*R*^*,eGFP*			This work Addgene ID 194083
**Primers**		**Sequence (5′–3′)**		**Source (usage)**
*cit* Z-M-F		ATGACAGCGACACGCGGTCTT		For PCR
*cit* Z-M-R		GGCTCTTTCTTCAATCGGAACGAAT		
*cit* Z-II-F		GCCTGCTTGACAGCGAGG		
*cit* Z-II-R		GGCTCTTTCTTCAATCGGAACGAAT		
*cit* Z-IX-F		AAGGGGTTGTAGCAACAACATCATCT		
*cit* Z-IX-R		GCGGATCAGACGGTTGTTGT		
V^7^_12(1)_-*BHQ*_2_*		TCTCTGTCATTTTTAATCATCGGATCCTAT-*BHQ*_2_		Labeled fluorophore
*Cy3-V*^4^_7(2)_		*Cy3*-ATTCTACGGATCCATGCTTA		
V^10^_12(1)_-Cy3^**^		CTACATTTTTACCCCTTGGATCCTG-*Cy3*		
V^7^_10_-*BHQ*_*2*_		AAGTGCGGGATCCCGCTCAA-*BHQ*_*2*_		

*For the *BHQ*_2_ quenching group, the absorption spectrum is 560–570 nm.

**The *Cy3* fluorophore group (excitation wavelength: ~550, emission wavelength: ~570 nm) can be excited using the 543.5 nm laser.

### Multimodular assembly reaction

A multimodular assembly method was used in this study. The structure of the *cit*Z-box was divided into nine modules with a 9984 nt repetitive scaffold strand. In an assembly reaction for each module, 25 ng of scaffold strands were mixed with 3.0 nM of the corresponding staple strands at a molar ratio of 1:10 in TAE/Mg^2+^ buffer (2 mol/L Tris, 0.1 mol/L CH_3_COOH, 0.05 mol/L EDTA, 12.5 mmol/L Mg(CH_3_COO)_2 _pH 8.0) to form a 20 μL assembly reaction system. The multimodular assembly reaction solutions were annealed in a thermocycler using the following temperature gradient: denaturation at 95 °C for 5 min, reduction to 65 °C at 0.6 °C per second, maintenance at 65 °C for 30 min, 50 °C for 30 min, 37 °C for 30 min, 22 °C for 30 min, and finally storage at 4 °C. The assembly solutions of each module were purified using gel electrophoresis and a TIANgel Midi purification kit to remove excess staple strands. Purified products of each module were remixed together at an equal ratio and annealed to hybridize with the shared staples again using the program: 95 °C for 5 min, reduction to 20 °C at 0.6 °C per second, and holding at 4 °C at the end. The yield of self-assembly was calculated according to the formula:${Y}=\frac{P}{k\times S}$, in which $Y$ is the yield of self-assembly; $k$ is the purification yield; $P$ is the weight of scaffolds used for assembling *citZ-*box after purification; and $s$ is the weight of the scaffolds as starting materials. The nucleic acid concentration was analyzed using Implen Microvolume Spectroscopy (Munich, Germany). The sequences of the DNA oligonucleotides used to form a single module are shown in Supplementary Tables S2–10, in which the inter-module shared staples are marked with gray shadowing and highlighted in red in the design schematic on the top of each table. The oligonucleotides used for assembly were synthesized by Ruibio Biotech. The TIANgel Midi purification kit and DNA ladder were purchased from TIANGEN Biotech.

### Plasmid/*eGFP* loading method

The purified samples of *cit*Z-boxes were taken from 4 °C or −20 °C storage and digested using *BamH*I restrictive enzyme (Takara). The enzyme reaction system included: 1.5 nM *cit*Z-boxes, 2 μL of *BamH*I (150 U), 10 μL of K buffer (10×), and ddH_2_O to a final volume of 50 μL, which was incubated at 30 °C for 60 min and held at 55 °C for 10 min to inactivate the restriction enzyme in a Tgradient thermocycler. Different plasmids were added into the digested *cit*Z-boxes at a molar ratio of 10:1. The final volume of the loading system was 50 μL, which included 1.5 nM digested *cit*Z-boxes, 15 nM plasmids, and ddH_2_O to 50 μL, which were incubated for 30 min at 37 °C. At the end of loading reaction, T_4_ ligase (700 U in 2 μL) was added into the solution and incubated at 22 °C overnight to seal and construct *cit*Z-boxes carrying plasmids: *GpCas9* (*Cm*^*R*^), *GpX461* (*Amp*^*R*^*, eGFP*), *GpY094* (*Amp*^*R*^*, eGFP*). Agarose gel electrophoresis was used to remove the excess plasmids and the loaded *cit*Z-boxes were purified using a gel purification kit. The plasmids used in this study included *pCas9* (catalog: 42876), *pX461* (catalog: 48140), and *pY094* (catalog: 84743) which were obtained from Addgene’s non-profit plasmid library (http://www.addgene.org). *Escherichia coli* DH5α and Top10 cultured in Luria–Bertani medium were used to host and amplify the plasmids (Table1).

### Plasmid/*eGFP* delivery method

*B. subtilis* 168 protoplasts were prepared by digestion with lysozyme^32^. *GpCas9*,*GpX461*, and*GpY094*were delivered into the protoplasts using the modified method of Chang and Cohen (1979)^33^. The *cit*Z-box carrying plasmids at 1.5 nM were mixed with 10^6^–10^8^ colony-forming units (cfu) of protoplasts and 1.5 μM polyethylene glycol (PEG) 4000 in a microtube at a final volume of about 550 μL. After gentle mixing, the mixture was incubated at 37 °C for 2 min in a water bath shaker to allow the plasmids to enter the protoplasts, and then 0.5 mL of SMMP (1.5 g of beef extract, 1.5 g of yeast extract, 5 g of peptone, 3.5 g of NaCl, 1 g of glucose, 3.68 g of K_2_HPO_4_, 1.32 g of KH_2_After_4_, 342.3 g of sucrose, 4.64 g of maleic acid, 8.12 g of MgCl_2_·6H_2_O, and deionized water to 1000 mL, pH 7.0) was added to terminate transformation. Protoplasts were harvested by centrifugation (10,000 × *g*, 7 min,) and the supernatant was discarded. SMMP (300 μL) was added and incubated for 60−90 min at 37 °C in a shaking water bath (100 rev/min) to allow for the expression of the resistance and *eGFP* genes. The solutions were then diluted appropriately with 1 × SMM (342.3 g of sucrose, 4.64 g of maleic acid, 8.12 g of MgCl_2_·6H_2_O, and deionized water to 1000 mL, pH 7.0,) and 0.1 mL was plated on complete medium for regeneration (CMR) (10 g of glucose, 10 g of peptone, 10 g of yeast extract, 5 g beef extract, 5 g of NaCl, 342.3 g of sucrose, 8.13 g of MgCl_2_, and deionzed water to 1000 mL, pH 7.2–7.5, and then 1.6% agar was added, before sterilization at 0.1 Mpa for 20 min) containing 25 μg/mL chloramphenicol for *GpCas9* or 100 μg/mL ampicillin for *GpX461* and *GpY094* to select transformants^34^. The experiments were repeated in triplicate to assay colony formation per μg of plasmid DNA added in the *cit*Z-box loading system, while the corresponding free plasmids at the same concentration were used as controls in the transformation experiments.

### AFM, SEM, and TEM imaging

When the last multimodular assembly reaction was completed, 500 μL of ethanol (95%) was added into the *cit*Z-box assembly solution and mixed uniformly, the sample was harvested by centrifugation (5000×*g*, 10 min) to remove salts, resuspended in 50 μL ddH_2_O, and then dispersed ultrasonically at room temperature for 20 min before imaging. Five microliters of appropriate diluted samples were deposited onto a pure 10 × 10 mm^2^ freshly cleaved mica and silicon surfaces, respectively, and incubated for 5 min. Then, the samples on mica were prepared for TEM imaging with uranyl acetate staining on a FEI-Tecnai G2 Spirit Twin (Hillsboro, OR, USA) instrument operated at 120 kV^35^. The samples on silicon were freeze-dried for 8 h and scanned in the SCANASYST-AIR mode using a Bruker ICON AFM (Berlin, Germany) with a cantilever length of 115 μm and a light spring constant of 0.4 N/m. After the engagement, the tapping amplitude set point was typically 0.5 V and the scan rates ranged from 0.6 to 1.0 Hz. The same silicon with the sample was gold-plated and observed using a Zeiss Merlin Compact scanning electron microscope (Jena, Thuringia, Germany). The working distance was 18.1 mm, and the detection voltage was 10.00 KV.

### Fluorescence resonance energy transfer (FRET)

To detect the can-lip opening process of *cit*Z-boxes, the labeled staple strands of *Cy3*-V^4^_7(2)_, V^10^_12(1)_-*Cy3*, V^7^_12(1)_-*BHQ*_*2*_, and V^7^_10_-*BHQ*_*2*_ (Table 1) with the *Cy3* fluorophore (excitation wavelength: ~550 nm, emission wavelength: ~570 nm) and *BHQ*_*2*_ dark quencher (absorption: 560–570 nm) were used to replace the staple strands of V^7^_12(1)_, V^4^_7(2)_, V^7^_10_ and V^10^_12(1)_ to assemble a fluorescent *cit*Z-box, named the *cit*Z-box^*Cy3-BHQ2*^. FRET spectroscopy measurements were used to monitor the opening of the *cit*Z-boxes in a Hitachi F-7000 fluorescence spectrophotometer (Tokyo, Japan). The aqueous solutions of *cit*Z-boxes were diluted 500-fold with ddH_2_O and detected from 400 nm to 700 nm at the excitation wavelength, *λ*_ex_ = 550 nm with a 150 w xenon lamps and a 10 nm slit in the emission channels, when the restriction enzyme *BamH*I and T_4_ ligase were added to open and close the *cit*Z-boxes, respectively.

### Confocal imaging and tracking *cit*Z-boxes in *B. subtilis*

*B. subtilis* 168 protoplasts transformed by *cit*Z-box^*Cy3-BHQ*^, *GpY094*, and ^*Cy3*^*G*^*pX461*::*eGFP*^ were incubated in SMMP medium, and then the protoplasts with the medium were moved to a 35 mm ThermoFisher cell dish (Waltham, MA, USA) and incubated at 37 °C for 240 min. During incubation, the status of the *cit*Z-boxes, as well as their loaded plasmids, was tracked and imaged using a Nikon A1 confocal laser-scanning microscope (Tokyo, Japan). Green and Red image channels were used to detect the fluorescence of eGFP and *Cy3,* respectively. We recorded the whole expression process of eGFP during the growth period of protoplasts using confocal laser-scanning microscopy (CLSM).

### Statistics and reproducibility

Quantitative data are presented as mean ± s.d. The dimensions of *cit*Z-box particles were calculated from dozens of AFM, SEM, and TEM images, selecting twenty particles per image. The purification yields of each module were means of ten replicates with three tests per sample. The transformation experiments were repeated in triplicate to assay the colonies formed by free plasmids and plasmid-loaded *cit*Z-boxes. The fluorescence intensity of eGFP and *Cy3* were detected in triplicate repeatedly using Green and Red image channels.

### Reporting summary

Further information on research design is available in the Nature Portfolio Reporting Summary linked to this article.

## Supplementary information

Supplementary Information

Description of Additional Supplementary Files

Supplementary data1

Supplementary data2

Supplementary data3

Supplementary data4

Supplementary data5

Supplementary Movie 1

Supplementary Movie 2

Supplementary Software

reporting summary

## Data Availability

The microscopy data, including the images of AFM, SEM, and TEM, were deposited in Image Data Resource, accession ID S-BIAD580. The uncropped and unedited gel images in Figs. 2b and  5b are included as Supplementary Figs. S2 and  S4. The plasmids-loaded DNA origami cubes generated in this study, *GpCas9* (194080), *GpX461* (194081), *GpY094* (194082), and ^*Cy3*^*G*^*pX461::eGFP*^ (194083) are deposited in Addgene. All source data underlying the graphs and charts in Figs. 3, 5b, 6b, 7b, and  8b are provided in Supplementary Data 1–5. All other data supporting the conclusions are included within the article and its Supplementary files.

## Code availability

The software package DEADALUS used is open-source and described in “Methods”. The modified DEADALUS code used to design the DNA origami cube are available for download at Zenodo^36^.
