# Supplementary material for: Self-assembled endogenous DNA nanoparticles for auto-release and expression of the eGFP gene in Bacillus subtilis
Source: Commun Biol. 2022 Dec 14;5:1373. doi: 10.1038/s42003-022-04233-8 (PMC9751278; doi:10.1038/s42003-022-04233-8)
Supplement: Supplementary file 1 — Supplementary Information [file 42003_2022_4233_MOESM1_ESM.pdf]

Supplementary Information

Self-assembled endogenous DNA nanoparticles for auto-release and expression of the *eGFP* gene in *Bacillus subtilis*

Linfeng Cao[1,2 §], Ziwen Meng[1,2 §], Junjie Tan[1,2 §], Ming Ying[1,2*], Meiying Bi[1,2], Yanjun Liu[1,2], Xinrui Tong[1,2], Jiaxun Wei[1,2], Lei Huang[1,2*]

[§]Equal contribution

Author's affiliations

[1] Tianjin Key Laboratory of Organic Solar Cells and Photochemical Conversion, School of Chemistry and Chemical Engineering, Tianjin University of Technology, Tianjin, China.

[2] Tianjin Key Laboratory of Drug Targeting and Bioimaging, School of Chemistry and Chemical Engineering, Tianjin University of Technology, Tianjin, China.

[*]Corresponding authors

Ming Ying, PhD
Email: ym@tjut.edu.cn

Lei Huang, PhD
Email: huanglei@tjut.edu.cn

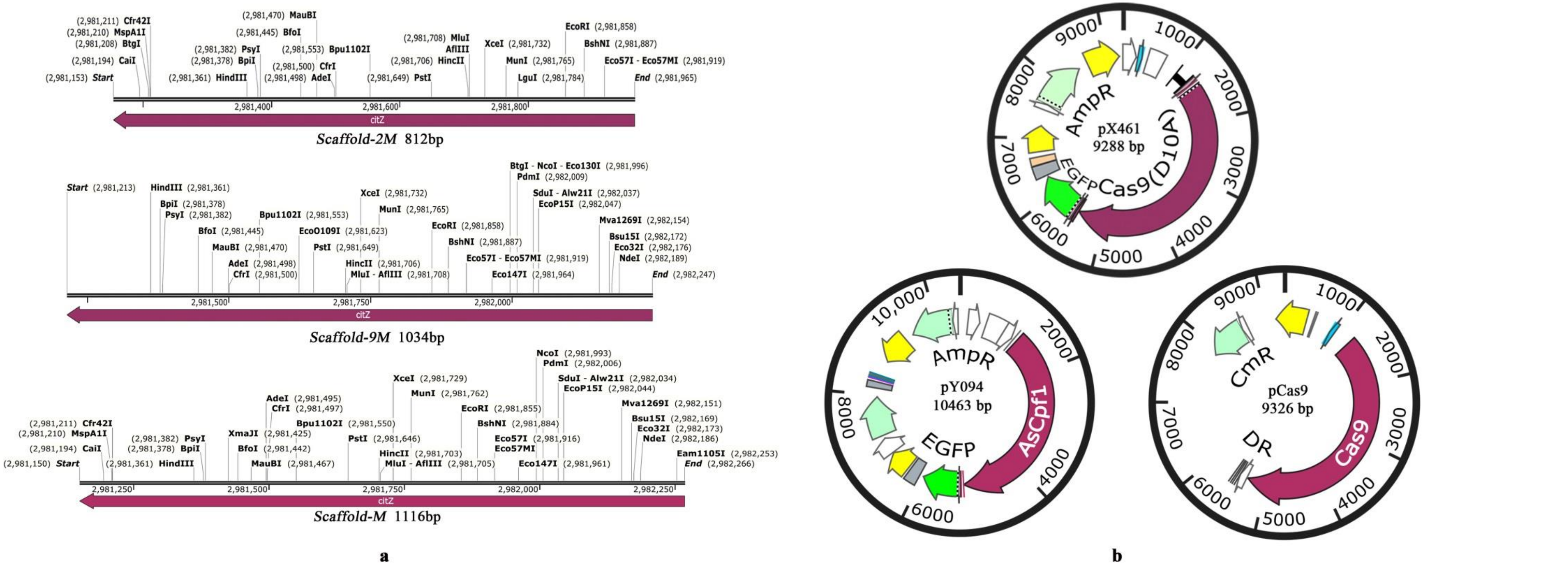

Fig. S1. The biological materials used in this experiment. **(a)** The *cit*Z gene maps used for scaffold strands with the restriction sites detected by Snapgene including *Scaffold-2M* (812 bp): the main scaffold of module II, *Scaffold-9M* (1034 bp): the main scaffold of module IX, and *Scaffold-M* (1116 bp): the scaffold of the remaining seven modules. **(b)** The annotated plasmid maps of *pX461*, *pY094*, and *pCas9*. Plasmids *pX461* and *pY094* carry the enhanced green fluorescent gene (*eGFP*) and the ampicillin resistance gene, Amp^R. Plasmid *pCas9* carries the chloramphenicol resistance gene, Cm^R.

Table S1. Sequences of the scaffold strands

Name	Position	Sequence (5′-3′)
	1–50	ATGACAGCGACACGCGGTCTTGAAGGGGTTGTAGCAACAACATCATCTGT
	51–100	TAGTTCTATTATTGATGATACCCTTACATATGTGGGGTATGATATCGATG
	101–150	ATTTGACAGAGAATGCAAGTTTTGAAGAAATCATCTACTTGCTTTGGCAT
	151–200	CTAAGGCTGCCAAACAAAAAAGAGCTTGAAGAGTTAAAGCAGCAGCTTGC
	201–250	AAAGGAAGCTGCCGTTCCGCAAGAAATTATTGAGCACTTCAAATCTTATT
	251–300	CCCTCGAAAATGTTCATCCCATGGCTGCTCTTCGGACAGCAATTTCATTA
	301–305	CTAGGCCTGCTTGACAGCGAGGCTGATACCATGAACCCGGAAGCCAACTA
	351–400	CAGAAAGGCGATCCGCCTTCAGGCAAAGGTGCCTGGCCTGGTTGCAGCGT
	401–450	TTTCCAGAATTCGCAAGGGTCTTGAGCCTGTTGAACCGAGAGAAGATTAC
	451–500	GGTATTGCGGAGAATTTTCTTTACACATTAAACGGTGAAGAGCCTTCACC
	501–550	AATTGAAGTTGAAGCCTTCAACAAAGCATTGATTTTACATGCTGACCATG
Scaffold-M	551–600	AGCTGAACGCGTCAACGTTCACAGCGAGGGTCTGCGTCGCAACACTTTCT
	601–650	GACATCTATTCAGGCATTACTGCAGCAATCGGCGCGCTGAAAGGGCCTCT
	651–700	TCACGGCGGAGCGAATGAAGGCGTTATGAAAATGCTGACAGAAATCGGTG
	701–750	AAGTGGAAAACGCTGAGCCGTACATCCGCGCTAAACTTGAAAAGAAAGAA
	751–800	AAAATCATGGGATTCGGCCACCGTGTGTATAAGCATGGTGATCCGCGCGC
	801–850	GAAGCATCTGAAAGAAATGAGCAAGCGCCTGACCAACCTGACAGGCGAAA
	851–900	GCAAATGGTATGAGATGTCGATTCGTATAGAAGACATCGTCACATCAGAG
	901–950	AAAAAGCTTCCGCCTAACGTTGATTTCTATTCGGCTTCTGTATATCACAG
	951–1000	CCTCGGTATTGACCATGATTTGTTTACACCGATTTTTGCTGTAAGCAGAA
	1001–1050	TGTCCGGCTGGCTCGCTCATATTCTTGAGCAGTATGACAACAACCGTCTG
	1051–1100	ATCCGCCCGCGGGCAGATTACACAGGCCCTGACAAACAAAAATTCGTTCC
	1101–1116	GATTGAAGAAAGAGCC
	305–354	GCCTGCTTGACAGCGAGGCTGATACCATGAACCCGGAAGCCAACTACAGA
	355–404	AAGGCGATCCGCCTTCAGGCAAAGGTGCCTGGCCTGGTTGCAGCGTTTTC
	405–454	CAGAATTCGCAAGGGTCTTGAGCCTGTTGAACCGAGAGAAGATTACGGTA
	455–504	TTGCGGAGAATTTTCTTTACACATTAAACGGTGAAGAGCCTTCACCAATT
	505–554	GAAGTTGAAGCCTTCAACAAAGCATTGATTTTACATGCTGACCATGAGCT
	555–604	GAACGCGTCAACGTTCACAGCGAGGGTCTGCGTCGCAACACTTTCTGACA
	605–654	TCTATTCAGGCATTACTGCAGCAATCGGCGCGCTGAAAGGGCCTCTTCAC
	655–704	GGCGGAGCGAATGAAGGCGTTATGAAAATGCTGACAGAAATCGGTGAAGT
Scaffold-2M	705–754	GGAAAACGCTGAGCCGTACATCCGCGCTAAACTTGAAAAGAAAGAAAAAA
	755–804	TCATGGGATTCGGCCACCGTGTGTATAAGCATGGTGATCCGCGCGCGAAG
	805–854	CATCTGAAAGAAATGAGCAAGCGCCTGACCAACCTGACAGGCGAAAGCAA
	855–904	ATGGTATGAGATGTCGATTCGTATAGAAGACATCGTCACATCAGAGAAAA
	905–954	AGCTTCCGCCTAACGTTGATTTCTATTCGGCTTCTGTATATCACAGCCTC
	955–1004	GGTATTGACCATGATTTGTTTACACCGATTTTTGCTGTAAGCAGAATGTC
	1005–1054	CGGCTGGCTCGCTCATATTCTTGAGCAGTATGACAACAACCGTCTGATCC
	1055–1104	GCCCGCGGGCAGATTACACAGGCCCTGACAAACAAAAATTCGTTCCGATT
	1105–1116	GAAGAAAGAGCC
Scaffold-2α	1–50	ATGACAGCGACACGCGGTCTTGAAGGGGTTGTAGCAACAACATCATCTGT

	51–67	TAGTTCTATTATTGATG
Scaffold-2β	1–50	ATACCCTTACATAGGATCC*GTATGGATCC*GATGATTTGACAGAGAATGCA
	51–89	AGTTTTGAAGAAATCATCTACTTGCTTTGGCATCTAAGG
Scaffold-2γ	1–50	CTGCCAAAGGATCC*AGAGGGATCC*GAGTTAAAGCAGCAGCTTGCAAAGGA
	51–84	AGCTGCCGTTCCGCAAGAAATTATTGAGCACTTC
Scaffold-2δ	1–50	AAATCTTATTCCCTCGAAAATGTTCATCCCATGGCTGCTCTTCGGACAGG
	51–64	ATCC*TCATGGATCC*
	23–72	AAGGGGTTGTAGCAACAACATCATCTGTTAGTTCTATTATTGATGATACC
	73–122	CTTACATATGTGGGGTATGATATCGATGATTTGACAGAGAATGCAAGTTT
	123–172	TGAAGAAATCATCTACTTGCTTTGGCATCTAAGGCTGCCAAACAAAAAAG
	173–222	AGCTTGAAGAGTTAAAGCAGCAGCTTGCAAAGGAAGCTGCCGTTCCGCAA
	223–272	GAAATTATTGAGCACTTCAAATCTTATTCCCTCGAAAATGTTCATCCCAT
	273–322	GGCTGCTCTTCGGACAGCAATTTCATTACTAGGCCTGCTTGACAGCGAGG
	323–372	CTGATACCATGAACCCGGAAGCCAACTACAGAAAGGCGATCCGCCTTCAG
	373–422	GCAAAGGTGCCTGGCCTGGTTGCAGCGTTTTCCAGAATTCGCAAGGGTCT
	423–472	TGAGCCTGTTGAACCGAGAGAAGATTACGGTATTGCGGAGAATTTTCTTT
	473–522	ACACATTAAACGGTGAAGAGCCTTCACCAATTGAAGTTGAAGCCTTCAAC
Scaffold-9M	523–572	AAAGCATTGATTTTACATGCTGACCATGAGCTGAACGCGTCAACGTTCAC
	573–622	AGCGAGGGTCTGCGTCGCAACACTTTCTGACATCTATTCAGGCATTACTG
	623–672	CAGCAATCGGCGCGCTGAAAGGGCCTCTTCACGGCGGAGCGAATGAAGGC
	673–722	GTTATGAAAATGCTGACAGAAATCGGTGAAGTGGAAAACGCTGAGCCGTA
	723–772	CATCCGCGCTAAACTTGAAAAGAAAGAAAAAATCATGGGATTCGGCCACC
	773–822	GTGTGTATAAGCATGGTGATCCGCGCGCGAAGCATCTGAAAGAAATGAGC
	823–872	AAGCGCCTGACCAACCTGACAGGCGAAAGCAAATGGTATGAGATGTCGAT
	873–922	TCGTATAGAAGACATCGTCACATCAGAGAAAAAGCTTCCGCCTAACGTTG
	923–972	ATTTCTATTCGGCTTCTGTATATCACAGCCTCGGTATTGACCATGATTTG
	973–1022	TTTACACCGATTTTTGCTGTAAGCAGAATGTCCGGCTGGCTCGCTCATAT
	1023–1056	TCTTGAGCAGTATGACAACAACCGTCTGATCCGC
Scaffold-9α	1–22	ATGACAGGATCC*CGCGGGATCC*

*The underlined positions are the *BamH*I sites.

Table S2. Sequences of staple strands of module I

Schematic

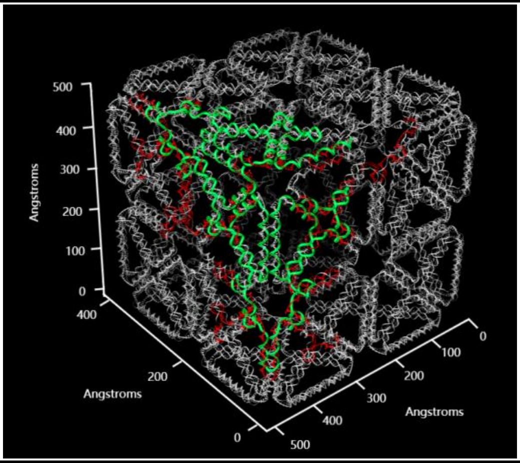

Name	Position	Sequence (5′-3′)
$V^{1}_{2(1)}$	I–IX	GGCCTTTTTTGTGTAATCTGTAGAAATCAACTTTTTGTTAGGC<br>GGATTGCTGCAGTATTTTTATGCCTGAATTAAAGA$_{78}$
$V^{1}_{2(2)}$	I–IX	TGAGCGAGCCCAATCGGAACGAATTTTTGTTGCTCAAGAATA$_{42}$
V^{1}_{4}	I	CCCGCGGGCGAGAAGCCGAA$_{20}$
$V^{4}_{6(1)}$	I	GAGGCTTTTTTGTGATATACGATCAGACGGTTTTTTTGTTGTCAT<br>AATATGA$_{52}$
$V^{4}_{6(2)}$	I	AATCAATACCATCTCTGGTC$_{20}$
V^{1}_{36}	I	TCTCGGTTCATTTTTTGTTTGGCAGCCTTAGCCGTAATCTTC$_{40}$
$V^{36}_{46(1)}$	I	TAACTTTTTTCTTCAAGCTCACAGGCTCAAGTTTTTACCCTTGC<br>GAACGCTGCAACCTTTTTAGGCCAGGCAAAGGCG$_{78}$
$V^{36}_{46(2)}$	I	TTGCAATCATTCGATAGCTG$_{20}$
$V^{31}_{36(1)}$	I–IX	AGCAGTTTTTCCATGGGATGAGGGAATAAGATTTTTTTTGAAGT<br>GCCTTGCGGAACGTTTTTGCAGCTTCCTCTGCTT$_{78}$
$V^{31}_{36(2)}$	I–IX	ATTGCTGTCTGTCAAGCAGGCCTATAGCGAAGTTGTAATGAA$_{42}$
$V^{35}_{36(1)}$	I–IX	TCCGCTTTTTCGTGAAGAGGCATTTCTTTCATTTTTGATGCTTCG<br>CCCATGC$_{52}$
$V^{35}_{36(2)}$	I–IX	TAACGCTTCCGTTGGCCTTC$_{20}$
$V^{36}_{37(1)}$	I–IX	GATCGTTTTTCCTTTCTGTAGGGTTCATGGTTTTTTATCAGCCTCGCCGAAG$_{52}$
$V^{36}_{37(2)}$	I–IX	GCGACGCACCTGAATAGATGTCAGGCCTGCCTTTAAAGTGTT$_{42}$
V^{36}_{40}	I	ATTCTACTTCCTTCAGGAAA$_{20}$
V^{6}_{37}	I–IX	TCAATTGGAATGCTTTGTTGAAGGCCACTGTTTTCTTCAACT$_{42}$
$V^{37}_{40(1)}$	I–IX	TCAGCTTTTTATGTAAAATCTGAAGGCTCTTTTTTTCACCGTTTA<br>AGTCCGAAGAGCTTTTTAGCCATGGGAGGGCGG$_{78}$
$V^{37}_{40(2)}$	I–IX	ATGCTTTTTTTTGTTGAAGGAATTGGTGAAGTTTTTGCTCTTCAC<br>CAAGTGT$_{52}$
$V^{37}_{40(3)}$	I–IX	CGCGTATGTATCAGCTCAGC$_{20}$
V^{6}_{37}	I	TGATGTGACGGAGGCCCTTTCAGCGCGCCGAAGCTTTTTCTC$_{42}$
$V^{6}_{40(1)}$	I	TCGCTTTTTTCCGCCGTGAAATGTCTTCTATTTTTTACGAATCGA<br>CATTTGCTTTCGTTTTTCCTGTCAGGTCTTGCT$_{78}$
$V^{6}_{40(2)}$	I	TTCATTGAACCGCTGAACGC$_{20}$

$V^{1}_{40(1)}$	I	AAATTTTTTTCTCCGCAATAATGCCAAAGCATTTTTAGTAGATGA<br>TTCAGACGGTTGTTTTTTTGTCATACTTGTCAG$_{78}$
$V^{1}_{40(2)}$	I	TGCGATTTTTCGCAGACCCTGTTGACGCGTTTTTTTCAGCTCAT<br>GGAAATCA$_{52}$
$V^{1}_{40(3)}$	I	AGATGTCAGAGTTTAATGTG$_{20}$
$V^{46}_{48(1)}$	I–IX	AACAGTTTTTATGATGTTGTCCTTCAAGACCTTTTTGCGTGTCGC<br>TCTTGCA$_{52}$
$V^{46}_{48(2)}$	I–IX	CATCAGAAGCAGGCGATAAT$_{20}$
$V^{2}_{46(1)}$	I–IX	CAAATTTTTTCATGGTCAATGCGGAAGCTTTTTTTTTTCTCTGAT<br>GATGATTTTTTCTTTTTTTTCTTTTCAGAAGGC$_{78}$
$V^{2}_{46(2)}$	I–IX	CAAAACAACCTGCTAATCGG$_{20}$
$V^{1}_{46(1)}$	I–IX	TTCTCTTTTTTGTCAAATCAACCCCACATATTTTTTGTAAGGGTA<br>TAGAACT$_{52}$
$V^{1}_{46(2)}$	I–IX	TTCTTCAAAAGTCATGCGGA$_{20}$
V^{36}_{48}	I–IX	TACCATTTTCTTCTATACGAATCGAATTTTCAATACATCTCA$_{42}$
$V^{36}_{49(1)}$	I–IX	GCTTATTTTTTACACACGGTCTGCTGCTTTATTTTTACTCTTCAA<br>GTTTGGC$_{52}$
$V^{36}_{49(2)}$	I–IX	AACATCGGATGCGCGTTTCG$_{20}$
$V^{6}_{11(1)}$	I–IX	TGATTTTTTTTTTTCTTTCTAGCGCGGATGTTTTTTACGGCTCAG<br>CTCACCGATTTCTTTTTTGTCAGCATTCTTCAT$_{78}$
$V^{6}_{11(2)}$	I–IX	CGCCTTTCAGGCACCTTTGCCTGAGCCGACGGTGAGGCGGAT$_{42}$
$V^{6}_{39(1)}$	I–IX	CAGGCTTTTTTCAAGACCCTAGTTGGCTTCCTTTTTGGGTTCAT<br>GGGCTGTC$_{52}$
$V^{6}_{39(2)}$	I–IX	TTTCATCTCGTCTTCAGTTT$_{20}$
V^{6}_{9}	I–IX	CTTCGAAATTTAATGCGCGC$_{20}$

Table S3. Sequences of staple strands of module II

**Schematic**		
		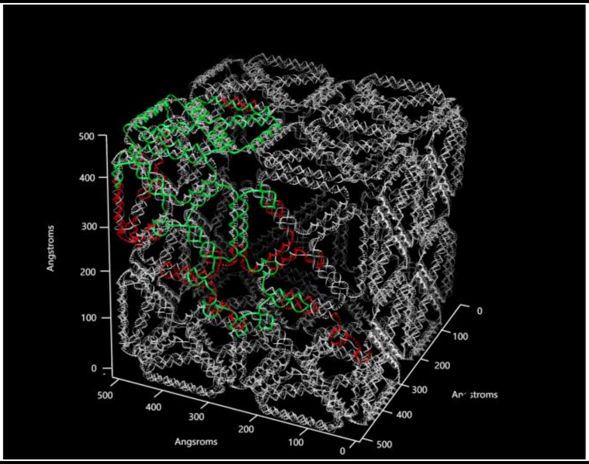

Name	Position	Sequence
V^{2}_{5}	II	TGAAGGCTCTGCCTGAAGGC$_{20}$
$V^{5}_{7(1)}$	II	AGGCCTTTTTAGGCACCTTTTCACCGTTTAATTTTTTGTGTAAA GACAATAC$_{52}$
$V^{5}_{7(2)}$	II	GGAAAGA$_{7}$GGATCC*A$_{14}$TACGCT$_{20}$
$V^{2}_{7(1)}$	II	GCTACTTTTTAACCCCTTCATCGCTGTCATGTTTTTGCTCTTTCT TAGCCGGACATTTTTTTCTGCTTACAGTGTAAA$_{78}$
$V^{2}_{7(2)}$	II	ATGGTATCAGATAATAGAACTAACAGATGATCTTCCGGGTTC$_{42}$
$V^{3}_{5(1)}$	II	CAGCTTTTTTCATGGTCAGCTCAGCGTTTTCTTTTTCACTTCACC GGCATTTTCATATTTTTACGCCTTCATGTGAAG$_{78}$
$V^{3}_{5(2)}$	II	CGTAATTTTTTCTTCTCTCGCTCAAGACCCTTTTTTTGCGAATTC TGCAACC$_{52}$
$V^{3}_{5(3)}$	II	TGAACCTCCGAAATTGTTGA$_{20}$
$V^{2}_{3(1)}$	II	TTCAATTTTTCTTCAATTGGGGATCGCCTTTTTTTTCTGTAGTTG GGTTGTT$_{52}$
$V^{2}_{3(2)}$	II	GATGTACGGCATGTAAAATCAATGCTTTGTTAGTTTAGCGCG$_{42}$
V^{2}_{47}	II	TGACGATGTCGCCGAATCCC$_{20}$
V^{16}_{47}	II	GTCATCTTGCAGGCGACTGC$_{20}$
$V^{47}_{48(1)}$	II	CCTGTTTTTTCAGGTTGGTCTCATTTCTTTCTTTTTAGATGCTTC GACCATG$_{52}$
$V^{47}_{48(2)}$	II	ATACCAATACTGGTCATTTG$_{20}$
$V^{3}_{47(1)}$	II	CTTATTTTTTACACACGGTGTTCTATACGAATTTTTTCGACATCT CCTTTCG$_{52}$
$V^{3}_{47(2)}$	II	ATTTCGGATCC*GCGCTGTCA$_{20}$
$V^{3}_{16(1)}$	II	AGGCCTTTTTCTTTCAGCGCGCAGTAATGCCTTTTTTGAATAGA TGTTGCGACGCAGTTTTTACCCTCGCTGCGCGTT$_{78}$
$V^{3}_{16(2)}$	II	GATCATTTTTGACGGTTGTTTCAAGAATATGTTTTTAGCGAGCC AGGATGCCAAAGCTTTTTAAGTAGATGAACTTGC$_{78}$
$V^{3}_{16(3)}$	II	TCTGCCCGTTTTGTTTGTCAGGGCCCGCCTCGCTCTGTGTAA$_{42}$

$V^{4}_{7(1)}$	I–II	GTAAGTTTTTGGTATCATCACCTCGCTGTCATTTTTAGCAGGC$_{43}$GGATCC*T$_{50}$GTCCGAATTTTTGA GCAGCCATTTTTCG$_{78}$
$V^{4}_{7(2)}$	I–II	ATTCTAC$_{7}$GGATCC*A$_{14}$TGCTTA$_{20}$
$V^{2}_{4(1)}$	I–II	GCGAGTTTTTCCAGCCGGACCAGCAAAAATCTTTTTGGTGTAAACAAATACC$_{52}$
$V^{2}_{4(2)}$	I–II	AGACCCAAGACTGCTGCGTG$_{20}$
$V^{6}_{7(1)}$	I–II	CATTTTTTTTCTTTCAGATGGGATCACCATGTTTTTCTTATACACAATCCCA$_{52}$
$V^{6}_{7(2)}$	I–II	AAACTTGCCAAAGCAAGTAGATGAAGGCGTGGTCTTTCTTCA$_{42}$
$V^{7}_{8(1)}$	II–IX	AGGGATTTTTATAAGATTTGTAATTTCTTGCTTTTTGGAACGGCAGAAGCTG$_{52}$
$V^{7}_{8(2)}$	II–IX	TCAGGTTGTCTCATACCATTTGCTGAACAGGGATTTCGCCTG$_{42}$
$V^{5}_{8(1)}$	II–IX	GCGGATTTTTTCACCATGCTTCTTTCTTTTCTTTTTAAGTTTAGC GTGTTGCGACGCTTTTTAGACCCTCGCGACGCG$_{78}$
$V^{5}_{8(2)}$	II–IX	GTTCAATGCTTTCAGACAGG$_{20}$
V^{3}_{8}	II–VIII–IX	TGATTTTTTATACACACGGTGGCCAAGTGTCAGAGAATCCCA$_{42}$
V^{3}_{15}	II–IX	GCCGATCTGTCGATTTTGCT$_{20}$
$V^{15}_{16(1)}$	II–IX	AGAGGTTTTTCCCTTTCAGCCTGCAGTAATGTTTTTCCTGAATA GAGCTCAG$_{52}$
$V^{15}_{16(2)}$	II–III	ATTCGTGGCTTGTCACTCCG$_{20}$
V^{16}_{48}	II–VIII	GCTTACAGCACTTTTTTGTTTGGCAGCCTTACCGGACATTCT$_{42}$

*The underlined positions are the *BamH*I sites.

Table S4. Sequences of staple strands of module III

Schematic

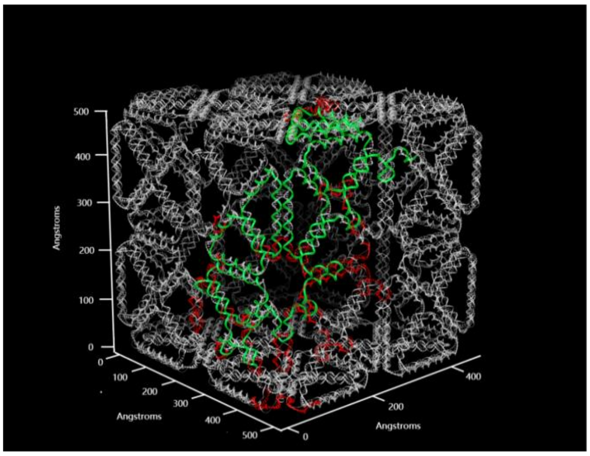

**Name**	**Position**	**Sequence (5′-3′)**
V^{16}_{20}	III	TCATCTGCCTACCTTAATAA$_{20}$
V^{16}_{21}	III	AATTGCTGGCTGTCAAGCAGGCCTTATCAATCGAAGTAATGA$_{42}$
$V^{20}_{21(1)}$	III	CAATATTTTTCCGTAATCTTAACAGGCTCAATTTTTGACCCTTGCGAACGCT$_{52}$
$V^{20}_{21(2)}$	III	AGTTGGCTTCTAAAGAAAAT$_{20}$
$V^{21}_{22(1)}$	III	TCACCTTTTTGTTTAATGTGCGGGTTCATGGTTTTTTATCAGCCTCTCCGAAGAGCATTTTTGCCAT GGGATGTCAGG$_{78}$
$V^{21}_{22(2)}$	III	TGCTTATACAGCTTCAACTTCAATTGGTGAAGCGGATCACCA$_{42}$
$V^{24}_{27(1)}$	III	CAGGGTTTTTCCTGTGTAATGCGGATCAGACTTTTTGGTTGTTGTCAGAATA$_{52}$
$V^{24}_{27(2)}$	III	ACAAATCATGACGAATTTTT$_{20}$
$V^{27}_{28(1)}$	III–IV	CTTTCTTTTTTTCAATCGGAGTCAATACCGATTTTTGGCTGTGATAGAAGCT$_{52}$
$V^{27}_{28(2)}$	III–IV	ATTGCTGTCCTCAAGACCGCGTGTCGCTGTCCTAGTAATGAA$_{42}$
V^{24}_{28}	III–IV	CTGCCCAGATACTAACGCGG$_{20}$
$V^{23}_{24(1)}$	VIII–III	GCGTGTTTTTTCGCTGTCATTCAATCGGAACTTTTTGAATTTTTGTCTGTGTAATCTTTTTTGCCCG CGGGCATATGA$_{78}$
$V^{23}_{24(2)}$	VIII–III	TGAGCTTTTTGAGCCAGCCGTTACAGCAAAATTTTTATCGGTGTAAGTTTGT$_{52}$
$V^{23}_{24(3)}$	VIII–III	CAACCGCTCAATACTCCTTC$_{20}$
V^{22}_{24}	III	TCTGTTCTGCGACATCAGCA$_{20}$
$V^{22}_{27(1)}$	III	TTTTCTTTTTTCTGATGTGATTTCCACTTCATTTTTCCGATTTCTGCCGTGAAGAGGTTTTTCCCTT TCAGCCTGCAG$_{78}$
$V^{22}_{27(2)}$	III	GTTAGGCGTACAGAAGCCGAATAGGCTCAGTACGAAATCAAC$_{42}$
$V^{22}_{25(1)}$	III	AAGTTTTTTTTAGCGCGGATGCGTTTTCCACTTTTTTTCACCGATTTTTTCATAACGTTTTTCCTTC ATTCGAAGAGG$_{78}$
$V^{22}_{25(2)}$	III	GTCAGTTTTTGTTGGTCAGGGTGGCCGAATCTTTTTCCATGATTTTTCAAGT$_{52}$
$V^{22}_{25(3)}$	III	TTTTTTGCTTCCATTTCTTT$_{20}$
V^{25}_{27}	III–V	GGCTCAGCGTCGATGTCTTC$_{20}$
$V^{16}_{50(1)}$	III	ATTCTTTTTTCTGTCAAATCTACCCCACATATTTTTTGTAAGGGTATAGAACTAACATTTTTGATGA TGTTGCCCTTC$_{78}$
$V^{16}_{50(2)}$	III	GGAACTTTTTGGCAGCTTCCATGTGACGATGTTTTTTCTTCTATACCTCATA$_{52}$
$V^{16}_{50(3)}$	III	TTTCTTAATTCTCAATCAAA$_{20}$
$V^{48}_{50(1)}$	III–V	TTAACTTTTTTCTTCAAGCTAAAATCGGTGTTTTTTAAACAATCACGAGGCTGTGATTTTTTATAC

		AGAAGAGCCGA$_{78}$
$V^{48}_{50(2)}$	III–V	TTTTTCTCTGTTTGCAAGCT$_{20}$
$V^{17}_{20(1)}$	III–VIII	CTTGCTTTTTGGAACGGCAGAAGCTGCTGCTTTTTTTTAACTCTTCTAGAAATCAACTTTTTGTTA GGCGGACTGATG$_{78}$
$V^{17}_{20(2)}$	III–VIII	GCAACTTTTTCAGGCCAGGCGAAGGCGGATCTTTTTGCCTTTCTGTTCTCCG$_{52}$
$V^{17}_{20(3)}$	III–VIII	AAGTGTGGAAAATTCCTCAA$_{20}$
V^{20}_{22}	III	CTCTCCATGTGTCAGGGTTC$_{20}$
$V^{17}_{22(1)}$	III–VIII	CGCGTTTTTTTCAGCTCATGAAAATCAATGCTTTTTTTTGTTGAAGCACGGTGGCCGTTTTTAATC CCATGACTTTTC$_{78}$
$V^{17}_{22(2)}$	III–VIII	GCTGTGAAGAAAGTGTTGCGACGCTTTGCCTTCCAGACCCTC$_{42}$
$V^{19}_{22(1)}$	III–VIII	TTTCTTTTTTTCAAAACTTGCAGCAAAAATCTTTTTGGTGTAAACAAATACC$_{52}$
$V^{19}_{22(2)}$	III–VIII	CAAAGCTGCAGATTGCAAGT$_{20}$
$V^{22}_{23(1)}$	III–VIII	CCCTTTTTTTTCAGCGCGCCGTAATGCCTGATTTTTATAGATGTCACGTTGA$_{52}$
$V^{22}_{23(2)}$	III–VIII	AATAGAACCACATATGTAAGGGTACCGTGCTCCGTCATCAAT$_{42}$

Table S5. Sequences of staple strands of module IV

Schematic

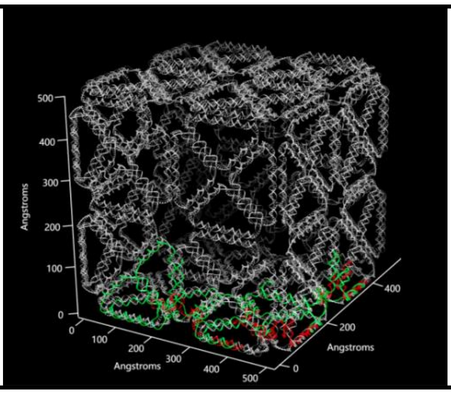

Name	Position	Sequence (5′-3′)
$V^{28}_{29(1)}$	IV	$AGATTTTTTTTGAAGTGCTCTGCGGAACGGCTTTTTAGCTTCCTTTGCTTTA_{52}$
$V^{28}_{29(2)}$	IV	$TAGTTTTTTTGGCTTCCGGGGACGCGTTCAGTTTTTCTCATGGTCATCAATG_{52}$
$V^{28}_{29(3)}$	IV	$CATTTTCGCCGCGGATCGAG_{20}$
V^{18}_{43}	IV	$TTATACACACCGAATTTTTGTTTGTCAGGGCGATCACCATGC_{42}$
$V^{18}_{42(1)}$	IV	$GATGCTTTTTTTCGCGCGCGCTGTGTAATCTTTTTTGCCCGCGGGCGTTGTTGTCATTTTTTACTGCTCAAGCAGCAA_{78}$
$V^{18}_{42(2)}$	IV	$TTGCTCAGCGCGGCTCATTT_{20}$
$V^{42}_{43(1)}$	IV	$TTTAGTTTTTCGCGGATGTATTTTCCACTTCTTTTTACCGATTTCTTCATAA_{42}$
$V^{42}_{43(2)}$	IV	$GAATATTTTTGATGTCAGAATGACGCGTTCATTTTTGCTCATGGTCATTGCTGTCCGTTTTTAAGAGCAGCCGAAGTG_{78}$
$V^{42}_{43(3)}$	IV	$TGCTGCAGTATTTCTTTCTT_{20}$
$V^{18}_{23(1)}$	IV–VII	$GCGAGTTTTTCCAGCCGGACCAAATCATCGATTTTTTATCATACCCTAACAGATGATTTTTTGTTGTTGCTAAAGACC_{78}$
$V^{18}_{23(2)}$	IV–VII	$GCTCAAGAGGATCAGACGGTTGTTTTTCGTTTGCGTCATACT_{42}$
$V^{23}_{42(1)}$	IV–VII	$CGCCTTTTTTTCATTCGCTCAGGCCCTTTCATTTTTGCGCGCCGATTTCAAG_{52}$
$V^{23}_{42(2)}$	IV–VII	$TTGTCCATTTGTCAGAGGGC_{20}$
$V^{23}_{28(1)}$	IV–VIII	$ATCATTTTTTCAATAATAGAGATGTTGTTGCTTTTTTACAACCCCTGAAGAGCAGCCTTTTTATGGGATGAAGGAATA_{78}$
$V^{23}_{28(2)}$	IV–VIII	$ACATATGTGTCAAATCATCGATATTTTCTGGCTCCATACCCC_{42}$
V^{28}_{42}	IV	$GTAGATGAAGCGCCGTGATT_{20}$
V^{28}_{43}	IV	$GTGAACGTAGTGTTGCGACGCAGATTTGGTTTTGCCCTCGCT_{42}$
$V^{28}_{41(1)}$	IV	$ACTCTTTTTTTCAAGCTCTTCAGCCTTAGATTTTTTGCCAAAGCAATCTTCAAAACTTTTTTTGCATTCTCTAAGGGT_{78}$
$V^{28}_{41(2)}$	IV	$GCAAGTTCAATCAACCTGCT_{20}$
$V^{28}_{33(1)}$	IV	$GAATTTTTTTCTGGAAAACGTGGTGAAGGCTTTTTTCTTCACCGTTAATCTTCTCTCTTTTTGGTTCAACAGCTTGCG_{78}$
$V^{28}_{33(2)}$	IV	$CTCAAGACGTAATCTTCTCTCGGTTTTCTAATAATCAACAGG_{42}$
V^{14}_{18}	IV–VII	$GCCTGTGGTCAATCATCAGG_{20}$
$V^{13}_{18(1)}$	IV–VII	$AAATCTTTTTGGTGTAAACAAATACCGAGGCTTTTTTGTGATATACGCTTTTTCTCTTTTTTGATGTGACGAACGAAT_{78}$
$V^{13}_{18(2)}$	IV–VII	$TCTGCTTAAATATGAGCGAGCCAGTACACGCTTACCGGACAT_{42}$

Table S6. Sequences of staple strands of module V

Schematic

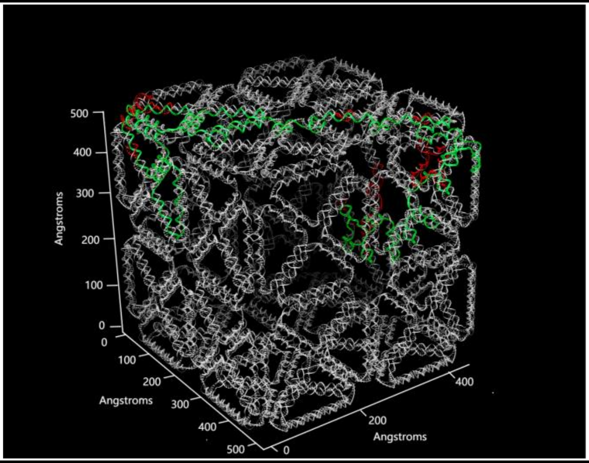

Name	Position	Sequence (5′-3′)
$V^{26}_{27(1)}$	V	GCCTGTTTTTTGTAATCTGCATCAGACGGTTTTTTTGTTGTCATACTCTGCT$_{52}$
$V^{26}_{27(2)}$	V	TCGCTCCGTCAGCATTTTCATAACTGTTTATTTTGCCTTCAT$_{42}$
$V^{25}_{26(1)}$	V	TTAGCTTTTTGCGGATGTACTATACGAATCGTTTTTACATCTCATATCGCCT$_{52}$
$V^{25}_{26(2)}$	V	TTCTTGGCGGCCGCGTCTTT$_{20}$
$V^{21}_{26(1)}$	V	TACAGTTTTTCAAAAATCGGTCATGGTCAATTTTTTACCGAGGCTGCATACCCCACATTT TTTATGTAAGGGAATAGA$_{78}$
$V^{21}_{26(2)}$	V	CCGGACATTGCTCAAGAATATGAGATGCTTTCAGCGAGCCAG$_{42}$
V^{21}_{25}	III–V	TATACACACGCGCTTGCTCA$_{20}$
$V^{26}_{50(1)}$	V	CCATTTTTTTTGCTTTCGCCGAGGGAATAAGTTTTTATTTGAAGTGTCTTGC$_{52}$
$V^{26}_{50(2)}$	V	TGTAAGACATGAATCACAAA$_{20}$
$V^{21}_{50(1)}$	III–V	CGCTTTTTTTGCTCATTTCTTCGCGCGCGGATTTTTTCACCATGCTTTTCTTTCAGATTTT TTGCTTCGCGCGGCTCT$_{78}$
$V^{21}_{50(2)}$	III–V	GAACATTTTCTGTCAGGTTG$_{20}$
V^{29}_{33}	V–IV	CAACTTCAATCTGCAACCAG$_{20}$
V^{27}_{29}	V–IV	TGTGAACGTTTTCATGGTAT$_{20}$
$V^{43}_{44(1)}$	IV–V	TTTCTTTTTTTCAATCGGAAGGTGGCCGAATTTTTTCCCATGATTTATGCCT$_{52}$
$V^{43}_{44(2)}$	IV–V	GATGATTTCTGTCGCTGTCA$_{20}$
$V^{41}_{43(1)}$	IV–V	GCTTTTTTTTGTTGAAGGCTTTGGTGAAGGCTTTTTTCTTCACCGTTTCTGT$_{52}$
$V^{41}_{43(2)}$	IV–V	TAGTAATGAAAGCATGTAAA$_{20}$
$V^{33}_{41(1)}$	IV–V	AGTTGTTTTTGCTTCCGGGTAGCCTCGCTGTTTTTTCAAGCAGGCCATCAAT$_{52}$
$V^{33}_{41(2)}$	IV–V	CGGATCGCCTTTAATGTGTA$_{20}$
$V^{18}_{44(1)}$	IV–V	CCTTCTTTTTAAGACCGCGTTCAAAACTTGCTTTTTATTCTCTGTCTATCAT$_{52}$
$V^{18}_{44(2)}$	IV–V	GGATCTTGCTTGTTGAGACG$_{20}$

Table S7. Sequences of staple strands of module VI

Schematic

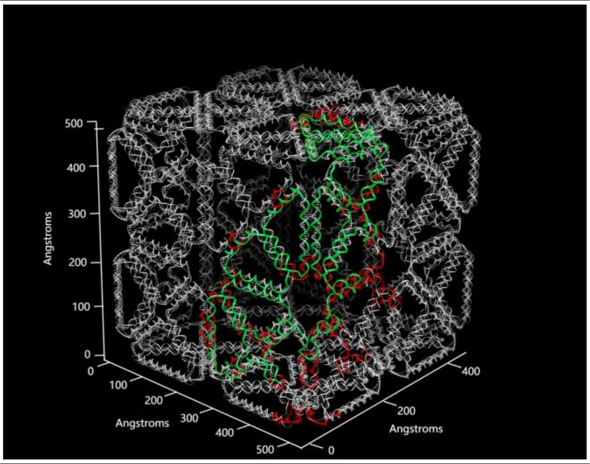

Name	Position	Sequence (5′-3′)
$V^{30}_{31(1)}$	VI	$CTGCATTTTTACCAGGCCAGCTGAAGGCGGATTTTTTCGCCTTTCTTCCGGG_{52}$
$V^{30}_{31(2)}$	VI	$CTGTCCGAAGCGAATTCTGG_{20}$
$V^{31}_{32(1)}$	VI	GGCTCTTTTTAAGACCCTTGAGCAGCCATGGTTTTTGATGAACATTTTTCTTGCGGATTTTT $ACGGCAGCTTCATGAT_{78}$
$V^{31}_{32(2)}$	VI	$ATGTACGGCTATACCGTAATCTTCTCTCGGTGTTTAGCGCGG_{42}$
V^{26}_{31}	VI	$CTCAATAATTCGAGGGAATAAGATCTACATGTTGTTGAAGTG_{42}$
V^{26}_{30}	V–VI	$GCTGTGTCAATCGCTCATGG_{20}$
$V^{27}_{30(1)}$	V–VI	TAATGTTTTTCCTGAATAGATGTTGCGACGCTTTTTAGACCCTCGCCAGCCTCGCTGTTTTT $TCAAGCAGGCATGGCT_{78}$
$V^{27}_{30(2)}$	V–VI	$TTCATTTTTTGGTATCAGCCGCAGGCCTAGTTTTTTAATGAAATTGAAAACG_{52}$
$V^{27}_{30(3)}$	V–VI	$GCGCCTGGCTGTAGTGATTG_{20}$
V^{30}_{32}	VI	$GCACCTTAATACCGTTTTGC_{20}$
$V^{27}_{32(1)}$	V–VI	TGGTGTTTTTAAGGCTCTTCGTGTAAAGAAATTTTTATTCTCCGCACAGCGTTTTCCTTTTT $ACTTCACCGACATTTT_{78}$
$V^{27}_{32(2)}$	V–VI	$GCTTCAACTGTAAAATCAATGCTTGAAAGTGTCATGTTGAAG_{42}$
$V^{32}_{33(1)}$	V–VI	$GCAGATTTTTCCCTCGCTGTGCGTTCAGCTCTTTTTATGGTCAGCATTCAAT_{52}$
$V^{32}_{33(2)}$	V–VI	$AATACCGTTAATGTGTAAAGAAAAAGTGTCAGAATTCTCCGC_{42}$
$V^{29}_{32(1)}$	V–VI	$CTTTGTTTTTTTGAAGGCTTGCCAGGCACCTTTTTTTTGCCTGAAGTTTCTG_{52}$
$V^{29}_{32(2)}$	V–VI	$GCATGTTGACGAACGTAAAA_{20}$
$V^{37}_{38(1)}$	VI–VII	$ATCAGTTTTTACGGTTGTTGTTTCTCTGATGTTTTTTGACGATGTCTTTCGC_{52}$
$V^{37}_{38(2)}$	VI–VII	$GAGGGAATAAGGGCCTGTGTAATCTGCCCGCTGAACATTTTC_{42}$
$V^{34}_{37(1)}$	VI	$TATGATTTTTGCGAGCCAGCGCTTACAGCAATTTTTAAATCGGTGTTGGTCA_{52}$
$V^{34}_{37(2)}$	VI	$GCGGAAGCTTTCATACTGCT_{20}$
$V^{33}_{34(1)}$	V–VI	AATTCTTTTTTGGAAAACGCCCAGGCACCTTTTTTTTGCCTGAAGGAAGAAAATTCTTTTTT $CCGCAATACCCCTTGC_{78}$
$V^{33}_{34(2)}$	V–VI	$ATACCTTTTTGAGGCTGTGACCGAATAGAAATTTTTTCAACGTTAGCAAGAA_{52}$
$V^{33}_{34(3)}$	V–VI	$GCTCAAATCAAAACAAGACC_{20}$
V^{34}_{38}	VI–VII	$CGGACCAATCTTCTTATTCT_{20}$
V^{32}_{34}	VI	$CCGATAGAAGTATACTGCTG_{20}$
$V^{32}_{37(1)}$	VI	CTGTCTTTTTAGGTTGGTCAGCGCCGATTGCTTTTTTGCAGTAATGGACCCTCGCTGTTTTT

		TGAACGTTGATCATGG$_{78}$
$V^{32}_{37(2)}$	VI	CCATTTGCTTCTATACGAATCGACCGCCGCGCTCATCTCATA$_{42}$
$V^{32}_{35(1)}$	VI	CATAATTTTTCGCCTTCATTTGAAGAGGCCCTTTTTTTTCAGCGCGCAGTAATGCCTTTTTT<br>GAATAGATGTTGCGAC$_{78}$
$V^{32}_{35(2)}$	VI	TTATATTTTTCACACGGTGGCGATTTCTGTCTTTTTAGCATTTTCAATTCGC$_{52}$
$V^{33}_{35(3)}$	VI	TTTCTGATCAGCGCGGTCAG$_{20}$
V^{35}_{37}	VI–IX	CCCTTTCAGCGGCGCTTGCT$_{20}$
V^{31}_{35}	VI–IX	TCCACTTCACCCGAATCCCA$_{20}$
$V^{26}_{49(1)}$	VI	ACTAATTTTTCAGATGATGTACCCCTTCAAGTTTTTACCGCGTGTCCTCTTTCTTCATTTTTA<br>TCGGAACGAGTCAGG$_{78}$
$V^{26}_{49(2)}$	VI	AGCCTTTTTTTAGATGCCAACTCATTTCTTTTTTTTCAGATGCTTCCACCAT$_{52}$
$V^{26}_{49(3)}$	VI	TATCATTTTGCTCTTTCAAT$_{20}$
V^{26}_{48}	V–VI	AGCCGAATAGTCTCTGTCAAATCATCGATATTGATATACAGA$_{42}$
$V^{48}_{49(1)}$	VI–IX	CTTCATTTTTAAACTTGCATAAATCAACGTTTTTTTAGGCGGAAGCGCTGCT$_{52}$
$V^{48}_{49(2)}$	VI–IX	CAGGCGCTTGAGCAAGTAGA$_{20}$

Table S8. Sequences of staple strands of module VII

Schematic

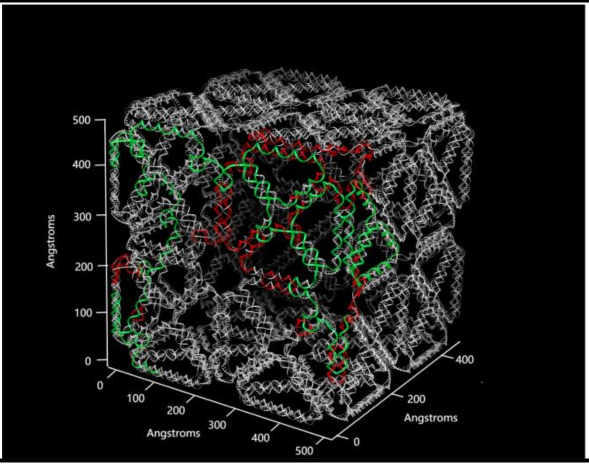

Name	Position	Sequence (5′-3′)
V^{38}_{41}	V–VII	TGTAAGTATCTCATGGGGTA$_{20}$
$V^{33}_{38(1)}$	V–VII	CGCTGTTTTTCATGGCTCTGGAACGAATTTTTTTTTGTTTGTCAGATTTGAAGTGTTTTTCTCAATAATTCGGCAG$_{78}$
$V^{33}_{38(2)}$	V–VII	TCAAGACCGATGATGTTGTTGCTACCAGGTGCAACAACCCCT$_{42}$
$V^{38}_{43(1)}$	V–VII	CTCAATTTTTTAATTTCTTGGCTTCCTTTGCTTTTTAAGCTGCTGCCCTTAGATGCCTTTTTAAAGCAAGTATGGCTC$_{78}$
$V^{38}_{43(2)}$	V–VII	TAAGATTTATGGGATGAACATTTTAAATCCTGTCCGAGGGAA$_{42}$
$V^{38}_{45(1)}$	VII	CAAAATTTTTCTTGCATTCTATCGATATCATTTTTTACCCCACATATCATCAATAATTTTTTAGAACTAACAGCGTGT$_{78}$
$V^{38}_{45(2)}$	VII	AAGTAATCTTCCGTAGATGA$_{20}$
$V^{12}_{43(1)}$	V–VII	GCCGATTTTTTTGCTGCAGTTAGATGTCAGATTTTTAAGTGTTGCGCTTAGATGCCATTTTTAAGCAAGTAGCAAAAC$_{78}$
$V^{12}_{43(2)}$	V–VII	TTTGGCAGTTTAACTCTTCAAGCTCCTTTGAGGCCTTTTTTG$_{42}$
$V^{43}_{45(1)}$	V–VII	AAATTTTTTTCTCCGCAATACTCTCGGTTCATTTTTACAGGCTCAAAAATCA$_{52}$
$V^{43}_{45(2)}$	V–VII	CGGAAATGTGGTTTACGGCA$_{20}$
$V^{11}_{12(1)}$	VII–IX	GCGTTTTTTTCAGCTCATGGAATTCTGGAAATTTTTACGCTGCAACACCTTTGCCTGTTTTTAAGGCGGATCGGAAAA$_{78}$
$V^{11}_{12(2)}$	VII–IX	GTTTGGCAGCACGCAGACCCTCGCTGTGAACAGCTCTTTTTT$_{42}$
V^{12}_{45}	VII	AATGCACTTCCTTCACTGAA$_{20}$
$V^{11}_{45(1)}$	VII	ATGCTTTTTTTTGTTGAAGGAATTGGTGAAGTTTTTGCTCTTCACCTAAAGA$_{52}$
$V^{11}_{45(2)}$	VII	TCAGCATGTAGACCCTTGCG$_{20}$
V^{11}_{38}	VII	TTTTGTTTGCTGCTTTAACTCTTCCAGGCCAGGCAAGCTCTT$_{42}$
$V^{38}_{39(1)}$	VII	CTTCCTTTTTTTTGCAAGCTGGCAGCCTTAGTTTTTATGCCAAAGCTTTCTT$_{52}$
$V^{38}_{39(2)}$	VII	AAGCATTTTTGGCCTAGTAAAAATTCTCCGCTTTTTAATACCGTAAGTTCAA$_{52}$
$V^{38}_{39(3)}$	VII	TCTTGGCCTCTATCACGGAA$_{20}$
$V^{11}_{39(1)}$	VII–IX	CGCTGTTTTTCAACCAGGCCTGTAGTTGGCTTTTTTTCCGGGTTCAAGCTGCTGCTTTTTTTTAACTCTTCAGTTGAC$_{78}$
$V^{11}_{39(2)}$	VII–IX	GCCTTTCTGTTGCGAATTCT$_{20}$
V^{19}_{23}	VII–VIII	CATTCTCTGTATTCTGCTTA$_{20}$
$V^{18}_{19(1)}$	IV–VII	CGACATTTTTTCTCATACCACCTGTCAGGTTTTTTTGGTCAGGCGCCTTTCA$_{78}$
$V^{18}_{19(2)}$	IV–VII	GAGGCTTTTTTGTGATATACTTGTTTGGCAGTTTTTCCTTAGATGCAGATGA$_{78}$

$V^{18}_{19(3)}$	IV–VII	$TGTCTTGGTCAATCATCTAT_{20}$
V^{17}_{19}	VII–VIII	$AAGCTCTTTTAGAAGCCGAA_{20}$
$V^{14}_{17(1)}$	VII–VIII	$ATACCTTTTTATTTGCTTTCTTGGTCAGGCGTTTTTCTTGCTCATTCGCAAT_{52}$
$V^{14}_{17(2)}$	VII–VIII	$CTGGAAAACGTACGAATCGA_{20}$
$V^{17}_{18(1)}$	IV–VII	$TGACGTTTTTATGTCTTCTACTGCAACCAGGTTTTTCCAGGCACCTGCTTCC_{52}$
$V^{17}_{18(2)}$	IV–VII	$AGGCGGAAAGAAGCCGAATAGAAATTTCTAGCTTTCAACGTT_{42}$
$V^{12}_{13(1)}$	VII–VIII	$ATTTCTTTTTTGTCAGCATTCTTCATTCGCTTTTTTCCGCCGTGAACAGCGC_{52}$
$V^{12}_{13(2)}$	VII–VIII	AATCGTTTTTGTGTAAACAAATACCGAGGCTTTTTTGTGATATACACTTCACCGTTTTTTT<br>$TAATGTGTAAAGCTTCG_{78}$
$V^{12}_{13(3)}$	VII–VIII	$TTCTGCTTACTACGGCTCAGCGTTTTCCACTCCAGCCGGACA_{42}$
V^{13}_{44}	V–VII	$TTTTTTCATCGGGTACTTTC_{20}$
$V^{12}_{44(1)}$	V–VII	$ACCCCTTTTTACATATGTAAAATAATAGAACTTTTTTAACAGATGAACAACC_{52}$
$V^{12}_{44(2)}$	V–VII	$TTCATATCGAAAATCAACGC_{20}$

Table S9. Sequences of staple strands of module VIII

Schematic

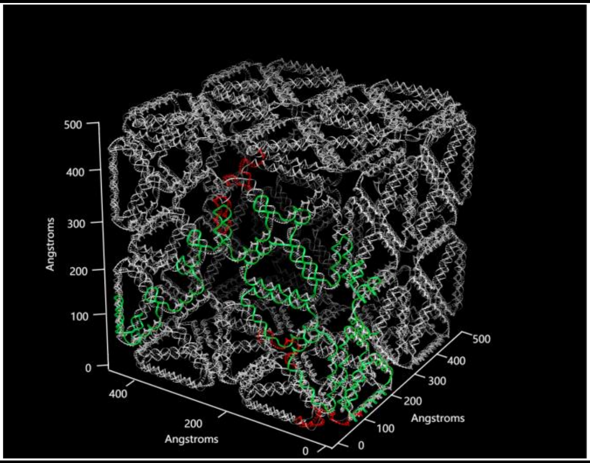

Name	Position	Sequence
V^{10}_{13}	VIII	GTGTAGGTCAATCATATCTG$_{20}$
V^{8}_{13}	VIII	GGTGAAGGCTGAAGCCGAATAGAAATCAACGCAACTTCAATT$_{42}$
$V^{8}_{10(1)}$	VIII	CTATATTTTTCGAATCGACAGTCAGGCGCTTTTTTTGCTCATTTCTTCGCGC$_{52}$
$V^{8}_{10(2)}$	VIII	TGTTTTTTTTGTCAGGGCCTCCCGCGGGCGGTTTTTATCAGACGGTTTGTTG$_{52}$
$V^{8}_{10(3)}$	VIII	GATGTGAACGAATCGGACGA$_{20}$
$V^{8}_{15(1)}$	VIII	CGTTTTTTTTTCCACTTCACCAGCATTTTCATTTTTTAACGCCTTCCCGTGA$_{52}$
$V^{8}_{15(2)}$	VIII	CGGATGTACGTGTCAGAAAG$_{20}$
$V^{15}_{17(1)}$	VIII	GCGCCGCTGTGCCTCGATTG$_{20}$
$V^{15}_{17(2)}$	III–VIII	AAGACTTTTTCGCGTGTCGCCTTTCTTCAATTTTTTCGGAACGAATCGGGCG$_{52}$
$V^{16}_{17(3)}$	III–VIII	GATGAACATTGCTGTCCGAAGAGCACAACTTGCTAGCCATGG$_{42}$
$V^{8}_{17(1)}$	VIII	TTCAGTTTTTCTCATGGTCATCAATGCTTTGTTTTTTTGAAGGCTTTTAGGCGGAAGTTTTT CTTTTTCTCTTGTCTT$_{78}$
$V^{8}_{17(2)}$	VIII	GGGTTTTTTTCATGGTATCACAAGCAGGCCTTTTTTAGTAATGAAATTTTCGAGGGATTTTT ATAAGATTTGTAATTT$_{78}$
$V^{8}_{17(3)}$	VIII	TGTAGTTGTTGCCTGAAGGCGGATACGTTTGTGACGCCTTTC$_{42}$
V^{8}_{14}	VIII	GCATGCAACACGGTTTAAAA$_{20}$
$V^{13}_{14(1)}$	VII–VIII	CGCGCTTTTTGGATCACCATACGGTGGCCGATTTTTATCCCATGATTTTTCAAGTTTTTTTT AGCGCGGATGAGCAAA$_{78}$
$V^{13}_{14(2)}$	VII–VIII	ACCGTTTTTTTAATCTTCTCTGGCTCAAGACCTTTTTCTTGCGAATTCATCTC$_{52}$
$V^{13}_{14(3)}$	VII–VIII	GAAAATTCTCTCTTTCAGAT$_{20}$

Table S10. Sequences of staple strands of module IX

Schematic

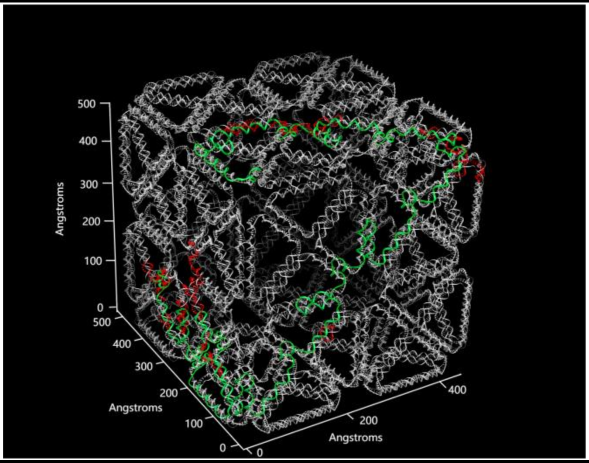

**Name**	**Position**	**Sequence (5′-3′)**
$V^{9}_{11(1)}$	IX	GTCAATTTTTGCAGGCCTAGGCTGTCCGAAGTTTTTAGCAGCCATGT TTCGA$_{52}$
$V^{9}_{11(2)}$	IX	TTCCTTTGCATGGTATCAGC$_{20}$
$V^{9}_{12(1)}$	IX	TTGCATTTTTTTCTCTGTCAGTATCATCAATTTTTTAATAGAACTACTGCTCAAGAATTTTT TATGAGCGAGTCACCG$_{78}$
$V^{9}_{12(2)}$	IX	AGTGCTTCTTATGATTCAAT$_{20}$
$V^{7}_{9(1)}$	II–IX	GGGAATTTTTTAAGATTTGAAATTTCTTGCGTTTTTGAACGGCAGCCTCGCT$_{52}$
$V^{7}_{9(2)}$	II–IX	TCCCTAACATGGATGCTGGA$_{20}$
$V^{7}_{12(1)}$	II–IX	CTGCTTTTTTTTAACTC$_{17}$GGATCC*T$_{24}$TTGGCAGTTTTTCCTTAGATGCATTCTCTGTCATTT TTAATCATC$_{69}$GGATCC*T$_{76}$AT$_{78}$
$V^{7}_{12(2)}$	II–IX	ATGTAAGGAATCATCGATATCATATTTGCCTTCCCCCCACAT$_{42}$
V^{7}_{10}	II–VIII–IX	AAGTGCGGGATCC*CGCTCAA$_{20}$
$V^{10}_{12(1)}$	VIII–IX	CTACATTTTTACCCCTT$_{17}$GGATCC*T$_{24}$GTCATGGTTTTTCTCTTTCTTCAATTTT$_{52}$
$V^{10}_{12(2)}$	VIII–IX	TGTTGTCATAACAGATGATG$_{20}$
$V^{2}_{48(1)}$	II–IX	ATAGATTTTTAATCAACGTTTTTTTCTCTGATTTTTTGTGACGATGGCTTTCGCCTGTTTTT TCAGGTTGGTTGATTT$_{78}$
$V^{2}_{48(2)}$	II–IX	GATATACAGACCGAATAGAAATCAACGTTAGACCGAGGCTGT$_{42}$
$V^{31}_{49(1)}$	VI–IX	TTTTTTTTTTCTTTCTTTTCCGGATGTACGGTTTTTCTCAGCGTTTTGATTTTTTCTTTTTTTT CTTTTCAATCAACA$_{78}$
$V^{31}_{49(2)}$	VI–IX	CCTTTGCAAGGGCCGAATCC$_{20}$
V^{37}_{39}	VII–IX	TGTGTAAAGATGAAATTGCT$_{20}$

*The underlined positions are *BamH*I sites.

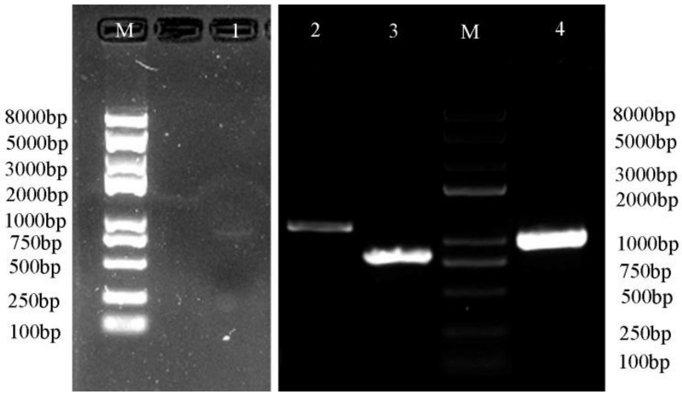

Fig. S2. Characterization of a complete *cit*Z-box and three types of scaffolds analyzed uaing agarose gel electrophoresis. 1: *cit*Z-boxes; 2: *Scaffold-M*; 3: *Scaffold-2M*; 4: *Scaffold-9M*; M: DNA marker.

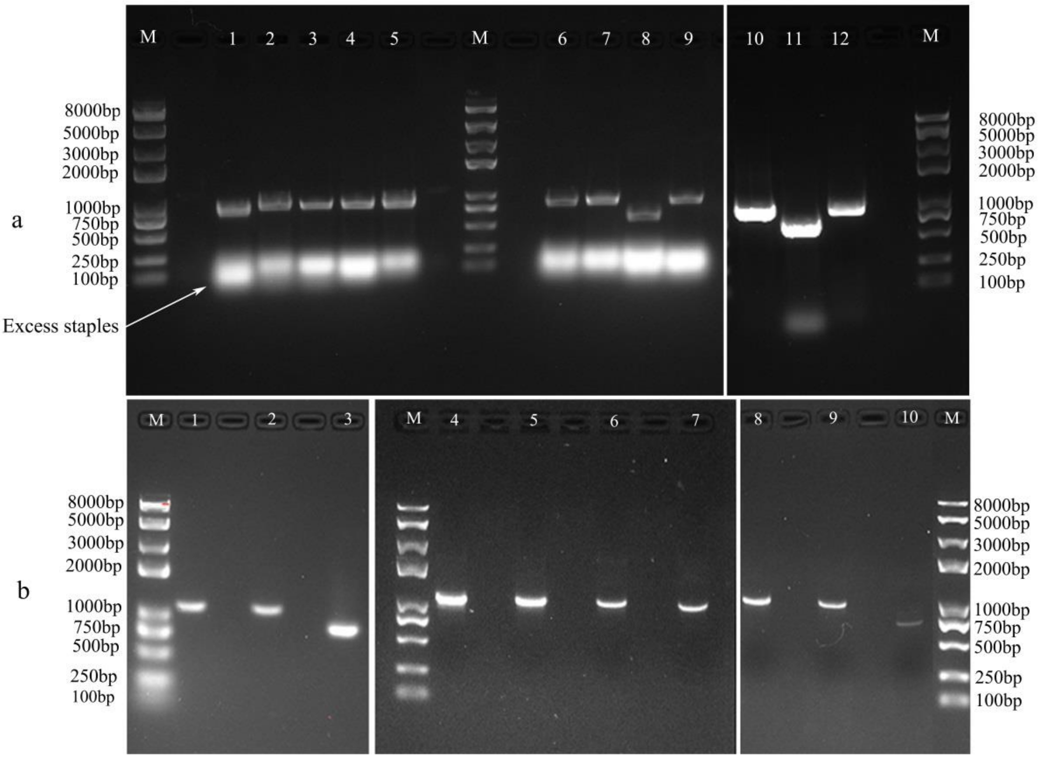

Fig. S3. Characterization of individual modules and *cit*Z-boxes analyzed using agarose gel electrophoresis. **(a)** The images of each module before purification and the scaffolds 1: Module IX; 2: Module VIII; 3: Module VII; 4: Module VI; 5: Module V; 6: Module IV; 7: Module III; 8: Module II; 9: Module I; 10: *Scaffold-9M*; 11: *Scaffold-2M*; 12: *Scaffold-M*; M: DNA marker. (b) The images of purified modules and the *cit*Z-boxes assembled directly 1: Module I; 2: Module IX; 3: Module II; 4: Module III; 5: Module IV; 6: Module V; 7: Module VI; 8: Module VII; 9: Module VIII; 10: *cit*Z-box; M: DNA marker.

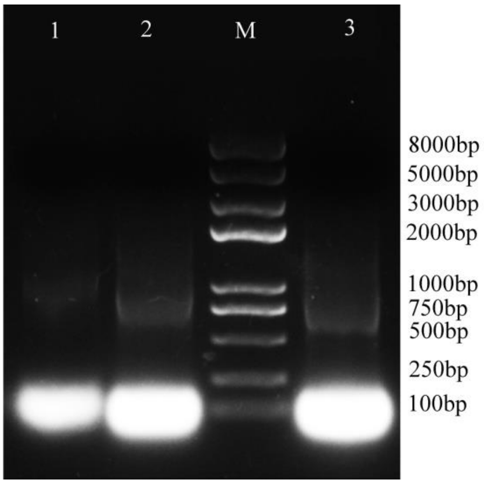

Fig. S4. Characterization of *cit*Z-boxes treated with *BamH*I and T_4 ligase analyzed using agarose gel electrophoresis.1: normal *cit*Z-box; 2: a *cit*Z-box opened by *BamH*I; M: DNA Marker, 3: a *cit*Z-box closed with T_4 ligase.

Table S11. Purification yields of individual modules

Characteristics	Individual modules								
	**I**	**II**	**III**	**IV**	**V**	**VI**	**VII**	**VIII**	**IX**
**Wpre-pur (ng)**	601.1 ± 4.3	759.05 ± 3.4	633.9 ± 2.1	639.1 ± 4.1	598.0 ± 4.0	597.9 ± 4.4	679.6 ± 4.3	605.4 ± 3.6	700.1 ± 3.6
**Wpost-pur (ng)**	241.1 ± 0.1	272.1 ± 0.1	303.0 ± 0.2	306.0 ± 0.6	271.5 ± 0.2	252.9 ± 0.6	289.5 ± 0.2	234.9 ± 0.5	249.9 ± 0.2
**Purification yields (%)**	40.1 ± 0.3	35.8 ± 0.1	47.8 ± 0.1	47.8 ± 0.2	45.4 ± 0.3	42.3 ± 0.2	42.6 ± 0.3	38.8 ± 0.1	35.7 ± 0.2

Data in the table are mean ± s.d. of ten replicates (with three tests per sample). **Wpre-pur**: the amount before purification; **Wpost-pur**: the amount after purification.
