# Supplementary material for: Self-assembled endogenous DNA nanoparticles for auto-release and expression of the eGFP gene in Bacillus subtilis
Source: Commun Biol. 2022 Dec 14;5:1373. doi: 10.1038/s42003-022-04233-8 (PMC9751278; doi:10.1038/s42003-022-04233-8)
Supplement: Supplementary file 2 — Description of Additional Supplementary Files [file 42003_2022_4233_MOESM2_ESM.pdf]

Description of Additional Supplementary Files

File name: Supplementary Movie 1

Description: An animated video showing the preparation of the citZ-boxes and the method to explore their function in host cells.

File name: Supplementary Movie 2

Description: This is a 4 h fast forwarded video tracked by CLSM that records the eGFP gene flowing out of the citZ-boxes and the expression process in B. subtilis protoplasts transformed by GpY094-loaded citZ-boxes.

File name: Supplementary Software

Description: The program coding package to design citZ-box.

File name: Supplementary Data 1

Description: The source data behind the graphs in Figure 3.

File name: Supplementary Data 2

Description: The source data behind the graphs in Figure 5b.

File name: Supplementary Data 3

Description: The source data behind the graphs in Figure 6b.

File name: Supplementary Data 4

Description: The source data behind the graphs in Figure 7b.

File name: Supplementary Data 5

Description: The source data behind the graphs in Figure 8b.
